# The Spectral Rrepresentation of a Discrete Version of Blackwell’s Markov Chain

**DOI:** 10.3390/e28050547

**Published:** 2026-05-11

**Authors:** Ernest Nieznaj

**Affiliations:** Department of Mathematics, Lublin University of Technology, Nadbystrzycka 38A, 20-618 Lublin, Poland; e.nieznaj@pollub.pl

**Keywords:** Markov chain, infinite state space, spectral representation, stationary distribution

## Abstract

We consider a Markov chain that can be termed a discrete version of Blackwell’s example from 1958. It is constructed with the aid of a sequence of independent Markov chains with two states. It turns out its stationary distribution π and transition matrix *P* are in detailed balance. As a result, the transition operator associated with *P* is self-adjoint in $\ell^{2}\left(\pi \right)$, the Hilbert space of all square summable sequences with respect to π. All eigenvalues of *P* are therefore real, and we give explicit formulae for them. Their corresponding eigenvectors form an orthogonal family in $\ell^{2}\left(\pi \right)$. Consequently, *P* can be diagonalized, and we find manageable formulae for $P^{n}$, where n≥2.

## 1. Introduction

In 1958, D. Blackwell provided an example of a continuous-time Markov chain with all states instantaneous (see [1] or more recent books [2,3]). The fact that all states of this process are instantaneous means its *Q*-matrix has −∞ in all its diagonal entries. This is one of the few such known examples; see [4] for another one. Some results related to this chain have been obtained recently (see [5,6]). As noted in these papers, the chain constructed by Blackwell is interesting because some explicit calculations can be carried out that are related to this process. For example, its stationary distribution was found in [6], where a stable case of this chain was also investigated.

In this paper we consider a discrete version of Blackwell’s example, which is constructed as follows. Let $X_{1}\left(n\right),X_{2}\left(n\right),\ldots$ be a sequence of independent Markov chains, each with two states 0 and 1. Our chain ${\left(X\left(n\right)\right)}_{n\geq 0}$ will be the joint process $\left(X_{1}\left(n\right),X_{2}\left(n\right),\ldots \right)$. Such a combination of independent Markov processes defines a new Markov process, whose state space is the set ${\left\{0,1\right\}}^{\infty }$ in our case. Details can be found in [7] (p. 45) and [3] (p. 65). Notice, however, that the set ${\left\{0,1\right\}}^{\infty }$ is uncountable. Therefore, in Lemma 2, we demonstrate that under certain conditions imposed on its components, ${\left(X\left(n\right)\right)}_{n\geq 0}$ takes values in a countable subset of ${\left\{0,1\right\}}^{\infty }$, which can be identified with the set of natural numbers N. In other words, the process formally defined by (28) will be a Markov chain on N. Its transition matrix *P* is determined by (30).

In Lemma 4, which is a generalization of [6] (Theorem 4), we find the stationary distribution of ${\left(X\left(n\right)\right)}_{n\geq 0}$. This distribution, denoted by π, is in detailed balance with *P*, which means Equation (39) hold true. As a result, the transition operator *T* associated with *P* (see (56) for definition) is a self-adjoint operator in $\ell^{2}\left(\pi \right)$, the Hilbert space of all sequences that are square summable with respect to π. This, in turn, implies that all eigenvalues of *T*, and therefore of *P*, are real. In Theorem 3, we give explicit formulae for these eigenvalues. Their corresponding eigenvectors form an orthogonal basis in $\ell^{2}\left(\pi \right)$ (see Theorem 4). Then, we can express *P* in the form $W^{-1}DW$, where *D* is a diagonal matrix with eigenvalues of *P* on its diagonal. The columns of *W* are eigenvectors corresponding to those eigenvalues. In Lemma 5, we show that $W^{-1}$ equals $c^{-1}W$, for a certain constant c>0. We finally obtain explicit formulae for $P^{n}=c^{-1}WD^{n}W$ for any n≥2 (see Examples 1 and 3). Such formulae, for a single two-state Markov chain, are available in many textbooks (see, e.g., [8,9]).

It is well known that transition matrices related to Markov chains can also be viewed as Markov operators in $l^{1}\left(N\right)$, the Banach space of all absolutely summable sequences (see [2] (p. 42)). Theorem 3 is formulated from this perspective. In consequence, the rows of *W* are vectors from $l^{1}\left(N\right)$.

It should also be added that there is no notion of an instantaneous state for a discrete-time Markov chain. Nevertheless, the Markov chain investigated here can be called a discrete version of Blackwell’s example because their construction is similar.

Probabilistic models using sequences of independent Markov chains with two or more states have some applications, especially in biology and medicine (see [10,11] and the references therein). For example, in [11], a model of *m* independent binary chains was studied. It has been used to detect genomic deletions in cancer patients. Statistical physics is another field where such models appear (see [3,12]). They are typically used to describe the behavior of independent particle systems.

The Markov chains studied in Section 2 and Section 3 may seem related to the Ising model, described in [13] (Chapter 7), as well as to birth and death processes (see [8] (p. 155)), whether with bounded or unbounded population. However, there are essential differences between them. The reason for this is the following. All entries of the transition matrix given by (5) or (30) are non-zero. The conclusion is that in the chains under study, each state is accessible from every other state in one step. Although a Markov chain used in the one-dimensional Ising model is defined on the state space ${\left\{-1,+1\right\}}^{m}$, which would be a case similar to the one considered in Section 2, not all transitions in one step are possible (see [13] (pp. 195–197)). Similarly, birth and death processes usually allow only for some, not all, transitions in one step (see [9] (pp. 185–187)).

The structure of this article is as follows. In Section 2, we begin with a Markov chain composed of a finite number of independent, two-state Markov chains. The notation introduced in this section, especially the binary representation of integers, will be used throughout the article. In Section 3, we consider a Markov chain with infinite state space. We find its stationary distribution and show that its state space can be identified with the set of natural numbers. Theorems 3 and 4 are the main results of this article. Theorem 1, being a special case of Theorem 3, is formulated in Section 2. Theorem 2 is equivalent to Theorem 1. Some additional results are included in Section 4.

## 2. The Spectral Representation of a $2^{m}$-State Markov Chain

The set of natural numbers {1,2,3,…} will be denoted by N. The set of all integers is denoted by Z. Additionally, in this section, $S_{m}$ stands for the set of integers from 1 to $2^{m}$, $S_{m}=\left\{1,2,3,\ldots ,2^{m}\right\}$.

Let an integer m≥2 be fixed and consider a Markov chain ${\left(X\left(n\right)\right)}_{n\geq 0}$ defined as

(1) $$X\left(n\right)≔\left(X_{1}\left(n\right),X_{2}\left(n\right),\ldots ,X_{m}\left(n\right)\right)\in {\left\{0,1\right\}}^{m},\;n\geq 0,$$

where $X_{1}\left(\cdot \right),\ldots ,X_{m}\left(\cdot \right)$ are mutually independent, homogeneous Markov chains, each with two states 0 and 1. As mentioned earlier, such a combination of independent chains defines another Markov chain (see [7] (p. 45)). The transition matrix of the component $X_{k}\left(\cdot \right)$ has then the form

(2) $$P_{k}=\left[\begin{array}{cc} 1-b_{k} & b_{k}\\ a_{k} & 1-a_{k} \end{array}\right]=\left[\begin{array}{cc} b_{k}^{\prime } & b_{k}\\ a_{k} & a_{k}^{\prime } \end{array}\right],$$

where, in general, $a_{k},b_{k}\in \left[0,1\right]$. In this paper we assume $a_{k},b_{k}\in \left(0,1\right)$ for k=1,…,m and denote also $a_{k}^{\prime }≔1-a_{k}$, $b_{k}^{\prime }:=1-b_{k}$. A homogeneous Markov chain is fully characterized by its transition matrix and initial distribution (see [8,9]). Thus, for the chain $X_{k}\left(\cdot \right)$ it would be $P_{k}$ and $X_{k}\left(0\right)$. The meaning of $a_{k}$ and $b_{k}$ in (2) is as follows:

(3) $$\left\{\begin{array}{c} P\left(X_{k}\left(n+1\right)=1|X_{k}\left(n\right)=0\right)=b_{k}\\ P\left(X_{k}\left(n+1\right)=0|X_{k}\left(n\right)=0\right)=1-b_{k}=b_{k}^{\prime }\\ P\left(X_{k}\left(n+1\right)=0|X_{k}\left(n\right)=1\right)=a_{k}\\ P\left(X_{k}\left(n+1\right)=1|X_{k}\left(n\right)=1\right)=1-a_{k}=a_{k}^{\prime } \end{array}\right.$$

for any n≥0, so these are the transition probabilities for $X_{k}\left(\cdot \right)$ in one step.

It is clear from the independence of its components that ${\left(X\left(n\right)\right)}_{n\geq 0}$ is also a homogeneous Markov chain, and in order to write its transition matrix, we will use the binary representation of integers introduced in [5,6]. This turned out to be a useful way of encoding natural numbers. We explain this representation here as well.

Basically, it all comes down to the fact that for every i∈N there exists a unique finite set $I_{i}\subset N$, such that

(4) $$i=1+\sum_{k\in I_{i}}2^{k-1},$$

where for i=1 we assume $I_{1}≔\emptyset$ and the sum over $I_{1}$ equals zero. For example, $I_{19}=\left\{2,5\right\}$ because $19=1+2^{2-1}+2^{5-1}$. The set $I_{i}$ in (4) we call the representation set of *i*. In this section, by $\bar{I_{i}}$ we denote the complement of $I_{i}$ with respect to {1,2,…,m}, i.e., $\bar{I_{i}}≔\left\{1,2,\ldots ,m\right\}\setminus I_{i}$. To summarize, we list representation sets of integers from 1 to 8: $I_{1}=\emptyset$, $I_{2}=\left\{1\right\}$, $I_{3}=\left\{2\right\}$, $I_{4}=\left\{1,2\right\}$, $I_{5}=\left\{3\right\}$, $I_{6}=\left\{1,3\right\}$, $I_{7}=\left\{2,3\right\}$, $I_{8}=\left\{1,2,3\right\}$.

**Lemma 1.**  *The set of states of* ${\left(X\left(n\right)\right)}_{n\geq 0}$*, defined by* (1)*, can be identified with* $S_{m}=\left\{1,2,\ldots ,2^{m}\right\}$*. Moreover, let* $P={\left(p_{ij}\right)}_{i,j\in S_{m}}$ *be its transition matrix. Then*

(5) $$p_{ij}=\prod_{k\in I_{i}\cap I_{j}}a_{k}^{\prime }\cdot \prod_{k\in I_{i}\cap \bar{I_{j}}}a_{k}\cdot \prod_{k\in \bar{I_{i}}\cap \bar{I_{j}}}b_{k}^{\prime }\cdot \prod_{k\in \bar{I_{i}}\cap I_{j}}b_{k},$$

*where* $I_{i}$*,* $I_{j}$ *are representation sets of i and j, respectively. We assume that if in* (5) *one of the sets* $I_{i}\cap I_{j}$*,* $I_{i}\cap \bar{I_{j}}$*,* $\bar{I_{i}}\cap \bar{I_{j}}$*,* $\bar{I_{i}}\cap I_{j}$ *is empty then the product over this set equals 1.*

**Proof.**  Let S denote the state space of ${\left(X\left(n\right)\right)}_{n\geq 0}$. Thus, it consists of all sequences $\left(\delta_{1},\delta_{2},\ldots ,\delta_{m}\right)$, where $\delta_{k}\in \left\{0,1\right\}$, for k=1,2,…,m. As explained in [6], a sequence $\delta =(\delta_{1},\delta_{2},\ldots ,\delta_{m})$ can be identified with the binary representation of an integer $i=1+\sum_{k=1}^{m}\delta_{k}2^{k-1}$. The smallest such integer equals 1, for the sequence (0,0,…,0), and the biggest equals $2^{m}$, for (1,1,…,1). In other words, we can define a map $f:S\to S_{m}$ in the following way

(6) $$f\left(\left(\delta_{1},\delta_{2},\ldots ,\delta_{m}\right)\right)=1+\sum_{k=1}^{m}\delta_{k}2^{k-1}.$$

Notice that *f* is a bijection because every natural number has a unique binary representation. We can rewrite (6) in an equivalent form f(δ)=i, where

$$S∋\delta =\left(\delta_{1},\delta_{2},\ldots ,\delta_{m}\right)\;⟷\;i=1+\sum_{k=1}^{m}\delta_{k}2^{k-1}\in S_{m}.$$

For example, for m=3 we have (0,0,0)→1, (1,0,0)→2, (1,1,0)→4, etc. The above observations justify that the set of states of ${\left(X\left(n\right)\right)}_{n\geq 0}$ can be regarded as $S_{m}$ and I assume we do so. Also, it can be seen from (4) and (6) that $I_{i}$ contains the indices of those components of δ which are equal to 1. Thus, $\bar{I_{i}}$ is the set of indices of δ which are equal to 0.

Fix $i,j\in S_{m}$ and let $I_{i}$, $I_{j}$ be their representation sets determined by (4). Then, denote $\delta ≔f^{-1}\left(i\right)$ and $\delta^{\prime }≔f^{-1}\left(j\right)$, where *f* is defined by (6). Because $I_{i}$ and $I_{j}$ are subsets of {1,2,…,m}, with possibility that $I_{i}=\emptyset$ and/or $I_{j}=\emptyset$, therefore

(7) $$\left\{1,2,\ldots ,m\right\}=\underset{1^{\prime }s\to 1^{\prime }s}{\underset{︸}{\left(I_{i}\cap I_{j}\right)}}\cup \underset{1^{\prime }s\to 0^{\prime }s}{\underset{︸}{\left(I_{i}\cap \bar{I_{j}}\right)}}\cup \underset{0^{\prime }s\to 0^{\prime }s}{\underset{︸}{\left(\bar{I_{i}}\cap \bar{I_{j}}\right)}}\cup \underset{0^{\prime }s\to 1^{\prime }s}{\underset{︸}{\left(\bar{I_{i}}\cap I_{j}\right)}}.$$

The meaning of the four pairwise disjoint sets in (7) is clear. They contain the indices of those components of δ which change to their corresponding components of $\delta^{\prime }$: from 1 to 1, from 1 to 0, from 0 to 0 and from 0 to 1. Hence, (5) follows from the independence of $X_{1}\left(\cdot \right),\ldots ,X_{m}\left(\cdot \right)$ and (3). □

As can be seen from (5), every $p_{ij}\in \left(0,1\right)$, and it is a product of exactly *m* numbers. This, in turn, implies that ${\left(X\left(n\right)\right)}_{n\geq 0}$ is irreducible (all states communicate) and aperiodic, which means that each state is accessible from every other state in one step.

Since the state space of ${\left(X\left(n\right)\right)}_{n\geq 0}$ is finite, $|S_{m}|=2^{m}$, there exists a unique stationary distribution $\pi ={\left(\pi_{i}\right)}_{i\in S_{m}}$ for this chain, which is determined by the equation π=πP; see, e.g., [9] (p. 133). In coordinates, it means

$$\pi_{j}=\sum_{i\in S_{m}}\pi_{i}p_{ij},\;j\in S_{m}.$$

In [6] (Theorem 4), it was proved that this distribution is given by

(8) $$\pi_{i}=\frac{1}{\prod_{k=1}^{m}\left(1+\frac{b_{k}}{a_{k}}\right)}\cdot \prod_{k\in I_{i}}\frac{b_{k}}{a_{k}},\;i\in S_{m}.$$

For example, for m=3 it takes the form

$$\pi =\frac{1}{\prod_{k=1}^{3}\left(1+\frac{b_{k}}{a_{k}}\right)}\cdot \left(1,\frac{b_{1}}{a_{1}},\frac{b_{2}}{a_{2}},\frac{b_{1}b_{2}}{a_{1}a_{2}},\frac{b_{3}}{a_{3}},\frac{b_{1}b_{3}}{a_{1}a_{3}},\frac{b_{2}b_{3}}{a_{2}a_{3}},\frac{b_{1}b_{2}b_{3}}{a_{1}a_{2}a_{3}}\right).$$

Let $l^{1}\left(R^{2^{m}}\right)$ denote $R^{2^{m}}$ with the norm defined by

$${∥x∥}_{l^{1}\left(R^{2^{m}}\right)}=\sum_{i=1}^{2^{m}}\left|\xi_{i}\right|,\;x={\left(\xi_{i}\right)}_{i\leq 2^{m}}=\left(\xi_{1},\xi_{2},\ldots ,\xi_{2^{m}}\right).$$

Then, the map x→x·P, where *P* is the transition matrix (5), is a Markov operator in $l^{1}\left(R^{2^{m}}\right)$ (see [2] (p. 42)). It means that ${∥x∥}_{l^{1}\left(R^{2^{m}}\right)}=∥x\cdot {P∥}_{l^{1}\left(R^{2^{m}}\right)}$ for any x≥0. By x≥0 we understand all components of the *x* are non-negative, i.e., $\xi_{i}\geq 0$.

Recall that a number λ is called an eigenvalue of *P* if there exists $x\in R^{2^{m}}$ such that x≠0 and xP=λx (see [14] (p. 134)). In such a case, *x* is termed an eigenvector of *P* corresponding to λ. In view of (8), it is clear that $\lambda_{1}=1$ is an eigenvalue of *P* and $x_{\lambda_{1}}=\pi$ is its corresponding eigenvector.

This theorem can be considered a generalization of the results from [8,9].

**Theorem 1.**  *The matrix* $P={\left(p_{ij}\right)}_{i,j\in S_{m}}$*, determined by* (5)*, has* $2^{m}$ *eigenvalues. These eigenvalues, denoted as* $\lambda_{1},\lambda_{2},\ldots ,\lambda_{2^{m}}$*, are given by*

(9) $$\lambda_{i}=\prod_{k\in I_{i}}\left(1-a_{k}-b_{k}\right)=\prod_{k\in I_{i}}\left(a_{k}^{\prime }-b_{k}\right),\;i\in S_{m}.$$

*The eigenvector* $x_{\lambda_{i}}=\left(\xi_{i,j}\right)$ *, corresponding to* $\lambda_{i}$*, equals*

(10) $$\xi_{i,j}={\left(-1\right)}^{|I_{j}\cap I_{i}|}\prod_{k\in I_{j}\cap \bar{I_{i}}}\frac{b_{k}}{a_{k}},\;j=1,2,\ldots ,2^{m}.$$

**Proof.**  See the proof of Theorem 3, where we consider a more general case. Those calculations also apply to this theorem. Another approach to the eigenvalues of *P* is presented in Theorem 2. □

**Remark 1.**  *Notice that* $\lambda_{1}=1$ *and* $|\lambda_{i}|<1$*, for* $i=2,3,\ldots ,2^{m}$*. This is because the Markov chain under study is irreducible and aperiodic (see [8] (p. 137)). We assume all* $a_{k},b_{k}\in \left(0,1\right)$*. However, not all these eigenvalues have to be distinct. For example, we may take* $a_{k}=b_{k}=\frac{1}{2}$*, for* k=1,2,…,m*. In this case, there are only two:* $\lambda_{1}=1$ *and* $\lambda_{2}=0$*.*

*Nevertheless, all vectors defined by* (10) *are linearly independent, and Theorem 1 allows us to diagonalize P. It can be written as*

(11) $$P=W^{-1}DW,$$

*where D and W are given by*

$$D=\left[\begin{array}{cccc} \lambda_{1} & 0 & \ldots & 0\\ 0 & \lambda_{2} & \ldots & 0\\ \ldots & \ldots & \ldots & \ldots \\ 0 & 0 & \ldots & \lambda_{2^{m}} \end{array}\right]\;W=\left[\begin{array}{c} x_{\lambda_{1}}\\ x_{\lambda_{2}}\\ \ldots \\ x_{\lambda_{2^{m}}} \end{array}\right]=\left[\begin{array}{cccc} \xi_{1,1} & \xi_{1,2} & \ldots & \xi_{1,2^{m}}\\ \xi_{2,1} & \xi_{2,2} & \ldots & \xi_{2,2^{m}}\\ \ldots & \ldots & \ldots & \ldots \\ \xi_{2^{m},1} & \xi_{2^{m},2} & \ldots & \xi_{2^{m},2^{m}} \end{array}\right]$$

*In Lemma 5, we prove that* $W^{-1}=c^{-1}\cdot W$*, where* $c=\prod_{k=1}^{m}\left(1+\frac{b_{k}}{a_{k}}\right)$*. Hence, from* (11)*, we would obtain*

(12) $$P^{n}=\frac{1}{\prod_{k=1}^{m}\left(1+\frac{b_{k}}{a_{k}}\right)}WD^{n}W,\;n\geq 1.$$

*Both* (11) *and* (12) *hold true also when D and W are infinite (see Theorem 3).*

**Example 1.**  *Consider* m=2*. The transition matrix of* $X\left(n\right)=(X_{1}\left(n\right),X_{2}\left(n\right))$ *has the form*

(13) $$P=\left[\begin{array}{cccc} b_{1}^{\prime }b_{2}^{\prime } & b_{1}b_{2}^{\prime } & b_{1}^{\prime }b_{2} & b_{1}b_{2}\\ a_{1}b_{2}^{\prime } & a_{1}^{\prime }b_{2}^{\prime } & a_{1}b_{2} & a_{1}^{\prime }b_{2}\\ b_{1}^{\prime }a_{2} & b_{1}a_{2} & b_{1}^{\prime }a_{2}^{\prime } & b_{1}a_{2}^{\prime }\\ a_{1}a_{2} & a_{1}^{\prime }a_{2} & a_{1}a_{2}^{\prime } & a_{1}^{\prime }a_{2}^{\prime } \end{array}\right]$$

*The four eigenvalues of P (see* (9)*) are as follows:*

$$D=\left[\begin{array}{cccc} \lambda_{1} & 0 & 0 & 0\\ 0 & \lambda_{2} & 0 & 0\\ 0 & 0 & \lambda_{3} & 0\\ 0 & 0 & 0 & \lambda_{4} \end{array}\right]=\left[\begin{array}{cccc} 1 & 0 & 0 & 0\\ 0 & a_{1}^{\prime }-b_{1} & 0 & 0\\ 0 & 0 & a_{2}^{\prime }-b_{2} & 0\\ 0 & 0 & 0 & \left(a_{1}^{\prime }-b_{1}\right)\left(a_{2}^{\prime }-b_{2}\right) \end{array}\right]$$

*and their corresponding eigenvectors are given by*

$$W=\left[\begin{array}{c} x_{\lambda_{1}}\\ x_{\lambda_{2}}\\ x_{\lambda_{3}}\\ x_{\lambda_{4}} \end{array}\right]=\left[\begin{array}{cccc} 1 & \frac{b_{1}}{a_{1}} & \frac{b_{2}}{a_{2}} & \frac{b_{1}b_{2}}{a_{1}a_{2}}\\ 1 & -1 & \frac{b_{2}}{a_{2}} & -\frac{b_{2}}{a_{2}}\\ 1 & \frac{b_{1}}{a_{1}} & -1 & -\frac{b_{1}}{a_{1}}\\ 1 & -1 & -1 & 1 \end{array}\right]$$

*In particular, for any* n≥2*, we have*

$$\left\{\begin{array}{c} p_{11}^{\left(n\right)}=\frac{1}{\prod_{k=1}^{2}\left(1+\frac{b_{k}}{a_{k}}\right)}\left(1+\frac{b_{1}}{a_{1}}\lambda_{2}^{n}+\frac{b_{2}}{a_{2}}\lambda_{3}^{n}+\frac{b_{1}b_{2}}{a_{1}a_{2}}\lambda_{4}^{n}\right)\\ p_{12}^{\left(n\right)}=\frac{1}{\prod_{k=1}^{2}\left(1+\frac{b_{k}}{a_{k}}\right)}\left(\frac{b_{1}}{a_{1}}\left(1-\lambda_{2}^{n}\right)+\frac{b_{1}b_{2}}{a_{1}a_{2}}\left(\lambda_{3}^{n}-\lambda_{4}^{n}\right)\right)\\ p_{13}^{\left(n\right)}=\frac{1}{\prod_{k=1}^{2}\left(1+\frac{b_{k}}{a_{k}}\right)}\left(\frac{b_{2}}{a_{2}}\left(1-\lambda_{3}^{n}\right)+\frac{b_{1}b_{2}}{a_{1}a_{2}}\left(\lambda_{2}^{n}-\lambda_{4}^{n}\right)\right)\\ p_{14}^{\left(n\right)}=\frac{1}{\prod_{k=1}^{2}\left(1+\frac{b_{k}}{a_{k}}\right)}\left(\frac{b_{1}b_{2}}{a_{1}a_{2}}\left(1-\lambda_{2}^{n}-\lambda_{3}^{n}+\lambda_{4}^{n}\right)\right). \end{array}\right.$$

*Taking the limit, we obtain*

$$\lim_{n\to +\infty }\left(p_{11}^{\left(n\right)},p_{12}^{\left(n\right)},p_{13}^{\left(n\right)},p_{14}^{\left(n\right)}\right)=\frac{1}{\prod_{k=1}^{2}\left(1+\frac{b_{k}}{a_{k}}\right)}\cdot \left(1,\frac{b_{1}}{a_{1}},\frac{b_{2}}{a_{2}},\frac{b_{1}b_{2}}{a_{1}a_{2}}\right),$$

*i.e., the stationary distribution for* X(n) *(see* (8)*).*

The transition matrix $P_{k}$, given by (2), has the following characteristic polynomial:

$$p\left(\mu \right)=\det\left(P_{k}-\mu I\right)=\left(\mu -1\right)\left(\mu -\left(1-a_{k}-b_{k}\right)\right),\;\mu \in C.$$

The eigenvalues of $P_{k}$, together with their corresponding left eigenvectors, are therefore equal

(14) $$\left\{\begin{array}{c} \mu_{1}^{k}=1,\;v_{1}^{k}=\left(1,\frac{b_{k}}{a_{k}}\right),\\ \mu_{2}^{k}=1-a_{k}-b_{k},\;v_{2}^{k}=\left(1,-1\right). \end{array}\right.$$

In other words, $v_{i}^{k}P_{k}=\mu_{i}^{k}v_{i}^{k}$, for i=1,2. Notice that each $\lambda_{i}$ (see (9)) is a product of $\mu_{i}^{k}$, for a suitable choice of *i*. This suggests that the transition matrix of the $2^{m}$-state Markov chain is the Kronecker product of the matrices $P_{1},\ldots ,P_{m}$. In Theorem 2, we prove that this is indeed the case, and we begin with the definition of the tensor product of matrices.

The tensor product or the Kronecker product of two matrices, $A=(a_{ij})$ and $B=(b_{ij})$, is defined to be the block matrix

$$A⊗B≔\left[\begin{array}{cccc} a_{11}B & a_{12}B & \ldots & a_{1n}B\\ a_{21}B & a_{22}B & \ldots & a_{2n}B\\ \ldots & \ldots & \ldots & \ldots \\ a_{m1}B & a_{m2}B & \ldots & a_{mn}B \end{array}\right]$$

See [15] (p. 243). So, if *A* is an m×n matrix and *B* is a p×q matrix, then A⊗B has mp rows and nq columns. Its (i,j) entry equals

(15) $${\left(A⊗B\right)}_{ij}=a_{f_{1}\left(i\right)f_{2}\left(j\right)}b_{g_{1}\left(i\right)g_{2}\left(j\right)},$$

where

$$\left\{\begin{array}{c} f_{1}\left(i\right)=\left(i-1\right)\;\mathrm{div}\;p+1,\;f_{2}\left(j\right)=\left(j-1\right)\;\mathrm{div}\;q+1,\\ g_{1}\left(i\right)=\left(i-1\right)\;\mathrm{mod}\;p+1,\;g_{2}\left(j\right)=\left(j-1\right)\;\mathrm{mod}\;q+1, \end{array}\right.$$

for i=1,2,…mp and j=1,2,…,nq. Here, the div notation stands for the usual integer division function: $i\;\mathrm{div}\;p≔\lfloor \frac{i}{p}\rfloor$, where ⌊·⌋ denotes the floor function (see [16] (p. 120)).

In general, A⊗B≠B⊗A; however, the tensor product is associative, i.e., (A⊗B)⊗C=A⊗(B⊗C), for any matrices. For example, $v_{1}^{1}⊗v_{1}^{2}$ and $v_{1}^{2}⊗v_{1}^{1}$ are

$$\left(1,\frac{b_{1}}{a_{1}}\right)⊗\left(1,\frac{b_{2}}{a_{2}}\right)=\left(1,\frac{b_{2}}{a_{2}},\frac{b_{1}}{a_{1}},\frac{b_{1}b_{2}}{a_{1}a_{2}}\right),\;\left(1,\frac{b_{2}}{a_{2}}\right)⊗\left(1,\frac{b_{1}}{a_{1}}\right)=\left(1,\frac{b_{1}}{a_{1}},\frac{b_{2}}{a_{2}},\frac{b_{1}b_{2}}{a_{1}a_{2}}\right).$$

The following theorem is equivalent to Theorem 1. It explains why $\lambda_{i}$s are products of $\mu_{i}^{k}$s, and also unveils the structure of $x_{\lambda_{i}}$s, their corresponding eigenvectors.

**Theorem 2.**  *Let* $P_{1},P_{2},\ldots ,P_{m}$ *and P be given by* (2) *and* (5)*, respectively. Then,*

(16) $$P=P_{m}⊗P_{m-1}⊗\ldots ⊗P_{2}⊗P_{1}.$$

*Moreover, any eigenvalue of P has the form*

(17) $$\mu_{i_{m}}^{m}\cdot \mu_{i_{m-1}}^{m-1}\ldots \mu_{i_{2}}^{2}\cdot \mu_{i_{1}}^{1}\;\left(\lambda_{i}=\prod_{k\in I_{i}}\mu_{2}^{k}\right),$$

*for some* $i_{1},i_{2},\ldots ,i_{m}\in \left\{1,2\right\}$*, and its corresponding eigenvector equals*

(18) $$v_{i_{m}}^{m}⊗v_{i_{m-1}}^{m-1}⊗\ldots ⊗v_{i_{2}}^{2}⊗v_{i_{1}}^{1}.$$

**Proof.**  In view of [15] (Theorem 4.2.12), the only thing that needs proof is (16). (17) and (18) follow from it. So we show (16) and the proof is by induction on *m*. We begin with m=2. This case does not require additional notation. The two matrices are

$$P_{2}=\left[\begin{array}{cc} b_{2}^{\prime } & b_{2}\\ a_{2} & a_{2}^{\prime } \end{array}\right],\;P_{1}=\left[\begin{array}{cc} b_{1}^{\prime } & b_{1}\\ a_{1} & a_{1}^{\prime } \end{array}\right],$$

so their Kronecker product equals

$$P_{2}⊗P_{1}=\left[\begin{array}{cc} b_{2}^{\prime }\cdot \left[\begin{array}{cc} b_{1}^{\prime } & b_{1}\\ a_{1} & a_{1}^{\prime } \end{array}\right] & b_{2}\cdot \left[\begin{array}{cc} b_{1}^{\prime } & b_{1}\\ a_{1} & a_{1}^{\prime } \end{array}\right]\\ a_{2}\cdot \left[\begin{array}{cc} b_{1}^{\prime } & b_{1}\\ a_{1} & a_{1}^{\prime } \end{array}\right] & a_{2}^{\prime }\cdot \left[\begin{array}{cc} b_{1}^{\prime } & b_{1}\\ a_{1} & a_{1}^{\prime } \end{array}\right] \end{array}\right]=\left[\begin{array}{cccc} b_{1}^{\prime }b_{2}^{\prime } & b_{1}b_{2}^{\prime } & b_{1}^{\prime }b_{2} & b_{1}b_{2}\\ a_{1}b_{2}^{\prime } & a_{1}^{\prime }b_{2}^{\prime } & a_{1}b_{2} & a_{1}^{\prime }b_{2}\\ b_{1}^{\prime }a_{2} & b_{1}a_{2} & b_{1}^{\prime }a_{2}^{\prime } & b_{1}a_{2}^{\prime }\\ a_{1}a_{2} & a_{1}^{\prime }a_{2} & a_{1}a_{2}^{\prime } & a_{1}^{\prime }a_{2}^{\prime } \end{array}\right],$$

which coincides with the matrix given by (13). The first step of induction is completed.

To continue the proof, let us introduce the following notation. The matrix $P={\left(p_{ij}\right)}_{i,j\in S_{m}}$ is now denoted $P\left(m\right)={\left(p_{ij}\left(m\right)\right)}_{i,j\in S_{m}}$. The matrix $P_{k}$ is now $P_{k}={\left(\alpha_{ij}\left(k\right)\right)}_{i,j\in \{1,2\}}$, i.e.,

(19) $$P_{k}=\left[\begin{array}{cc} \alpha_{11}\left(k\right) & \alpha_{12}\left(k\right)\\ \alpha_{21}\left(k\right) & \alpha_{22}\left(k\right) \end{array}\right]=\left[\begin{array}{cc} b_{k}^{\prime } & b_{k}\\ a_{k} & a_{k}^{\prime } \end{array}\right],\;k\geq 1.$$

Also, let $N_{m}≔\left\{1,2,\ldots ,m\right\}$, for m≥2. The second step of induction requires us to prove

(20) $$P\left(m+1\right)=P_{m+1}⊗P\left(m\right),$$

for m≥2. At first, we write explicit expressions for the entries of the two $2^{m+1}\times 2^{m+1}$ matrices in (20). Fix $i,j\in S_{m+1}=\left\{1,2,\ldots ,2^{m+1}\right\}$. Based on (15), we have

(21) $${\left(P_{m+1}⊗P\left(m\right)\right)}_{ij}=\alpha_{f_{1}\left(i\right)f_{2}\left(j\right)}\left(m+1\right)p_{g_{1}\left(i\right)g_{2}\left(j\right)}\left(m\right),$$

where

$$\left\{\begin{array}{c} f_{1}\left(i\right)=\left(i-1\right)\;\mathrm{div}\;2^{m}+1,\;f_{2}\left(j\right)=\left(j-1\right)\;\mathrm{div}\;2^{m}+1,\\ g_{1}\left(i\right)=\left(i-1\right)\;\mathrm{mod}\;2^{m}+1,\;g_{2}\left(j\right)=\left(j-1\right)\;\mathrm{mod}\;2^{m}+1. \end{array}\right.$$

Notice that $g_{1}\left(i\right),g_{2}\left(j\right)\in S_{m}$. This is because $\left(-1\right)\;\mathrm{mod}\;2^{m}=2^{m}-1$, which implies

$$\left(2^{m+1}-1\right)\;\mathrm{mod}\;2^{m}+1=2^{m}-1+1=2^{m}\in S_{m}.$$

The (i,j) entry of P(m+1) is

(22) $$p_{ij}\left(m+1\right)=\prod_{k\in I_{i}\cap I_{j}}a_{k}^{\prime }\cdot \prod_{k\in I_{i}\cap \bar{I_{j}}}a_{k}\cdot \prod_{k\in \bar{I_{i}}\cap \bar{I_{j}}}b_{k}^{\prime }\cdot \prod_{k\in \bar{I_{i}}\cap I_{j}}b_{k},$$

where $I_{i},I_{j}\subset N_{m+1}$. We will write $p_{ij}\left(m+1\right)$ in such a way that it can be directly compared with the expression on the right side of (21). To this end, let us define

$$I_{i}^{1}≔I_{i}\cap N_{m},\;I_{i}^{2}≔I_{i}\cap \left\{m+1\right\}\;\Rightarrow \;I_{i}=I_{i}^{1}\cup I_{i}^{2},\;I_{i}^{1}\cap I_{i}^{2}=\emptyset .$$

In particular, $I_{i}^{1}\subset N_{m}$ and $I_{i}^{2}\subset \left\{m+1\right\}$. In the latter case, $I_{i}^{2}=\emptyset$ or $I_{i}^{2}=\left\{m+1\right\}$. By direct calculation, we obtain the equalities

(23) $$\left\{\begin{array}{c} I_{i}\cap I_{j}=\left(I_{i}^{1}\cap I_{j}^{1}\right)\cup \left(I_{i}^{2}\cap I_{j}^{2}\right),\\ I_{i}\cap \bar{I_{j}}=\left(I_{i}^{1}\cap \bar{I_{j}^{1}}\right)\cup \left(I_{i}^{2}\cap \bar{I_{j}^{2}}\right),\\ \bar{I_{i}}\cap \bar{I_{j}}=\left(\bar{I_{i}^{1}}\cap \bar{I_{j}^{1}}\cap N_{m}\right)\cup \left(\bar{I_{i}^{2}}\cap \bar{I_{j}^{2}}\cap \left\{m+1\right\}\right),\\ \bar{I_{i}}\cap I_{j}=\left(\bar{I_{i}^{1}}\cap I_{j}^{1}\right)\cup \left(\bar{I_{i}^{2}}\cap I_{j}^{2}\right). \end{array}\right.$$

All complements in (22) and (23) are with respect to $N_{m+1}$. For example, the proof of the first equality is this

$$I_{i}\cap I_{j}=\left(I_{i}^{1}\cup I_{i}^{2}\right)\cap \left(I_{j}^{1}\cup I_{j}^{2}\right)=\left(I_{i}^{1}\cap \left(I_{j}^{1}\cup I_{j}^{2}\right)\right)\cup \left(I_{i}^{2}\cap \left(I_{j}^{1}\cup I_{j}^{2}\right)\right)=\left(I_{i}^{1}\cap I_{j}^{1}\right)\cup \left(I_{i}^{2}\cap I_{j}^{2}\right),$$

due to $I_{i}^{1}\cap I_{j}^{2}=\emptyset$ and $I_{i}^{2}\cap I_{j}^{1}=\emptyset$. Taking into account (23), the right hand side of (22) can be factorized as follows

(24) $$p_{ij}\left(m+1\right)=p_{ij}^{1}\left(m+1\right)p_{ij}^{2}\left(m+1\right),$$

where

$$\left\{\begin{array}{c} p_{ij}^{1}\left(m+1\right)≔\prod_{k\in I_{i}^{1}\cap I_{j}^{1}}a_{k}^{\prime }\cdot \prod_{k\in I_{i}^{1}\cap \bar{I_{j}^{1}}}a_{k}\cdot \prod_{k\in \bar{I_{i}^{1}}\cap \bar{I_{j}^{1}}\cap N_{m}}b_{k}^{\prime }\cdot \prod_{k\in \bar{I_{i}^{1}}\cap I_{j}^{1}}b_{k},\\ p_{ij}^{2}\left(m+1\right)≔\prod_{k\in I_{i}^{2}\cap I_{j}^{2}}a_{k}^{\prime }\cdot \prod_{k\in I_{i}^{2}\cap \bar{I_{j}^{2}}}a_{k}\cdot \prod_{k\in \bar{I_{i}^{2}}\cap \bar{I_{j}^{2}}\cap \left\{m+1\right\}}b_{k}^{\prime }\cdot \prod_{k\in \bar{I_{i}^{2}}\cap I_{j}^{2}}b_{k}. \end{array}\right.$$

Comparing (21) with (24), we conclude that to prove (20), we have to show

(25) $$p_{ij}^{1}\left(m+1\right)=p_{g_{1}\left(i\right)g_{2}\left(j\right)}\left(m\right),\;p_{ij}^{2}\left(m+1\right)=\alpha_{f_{1}\left(i\right)f_{2}\left(j\right)}\left(m+1\right).$$

$I_{i}^{1}$ and $I_{j}^{1}$ are subsets of $N_{m}$, as are $I_{i}^{1}\cap I_{j}^{1}$, $I_{i}^{1}\cap \bar{I_{j}^{1}}$, $\bar{I_{i}^{1}}\cap I_{j}^{1}$ and $\bar{I_{i}^{1}}\cap \bar{I_{j}^{1}}\cap N_{m}.$ So the proof of the first equality in (25) will follow from

(26) $$I_{g_{1}\left(i\right)}=I_{i}^{1}=I_{i}\cap N_{m},\;i\in S_{m+1}.$$

To substantiate it, we consider two cases. In the first one, let $i\leq 2^{m}$, i.e., $i\in S_{m}$. Then, $I_{i}\subset N_{m}$, so $I_{i}^{1}=I_{i}$, and

$$g_{1}\left(i\right)=\left(i-1\right)\;\mathrm{mod}\;2^{m}+1=i\;\Rightarrow \;I_{g_{1}\left(i\right)}=I_{i}.$$

In the second case, $i\in S_{m+1}\setminus S_{m}$, i.e., $i\in \{2^{m}+1,\ldots ,2^{m+1}\}$. Then, certainly $\left\{m+1\right\}\subset I_{i}$, which implies $I_{i}^{1}=I_{i}\cap N_{m}=I_{i}\setminus \left\{m+1\right\}$. On the other hand, $2^{m}\;\mathrm{mod}\;2^{m}=0$ results in

$$g_{1}\left(i\right)=i-2^{m}=i-2^{(m+1)-1}\;\Rightarrow \;I_{i-2^{m}}=I_{i}\setminus \left\{m+1\right\}.$$

Thus, (26) is proved. The last thing to demonstrate is

(27) $$\alpha_{f_{1}\left(i\right)f_{2}\left(j\right)}\left(m+1\right)=\prod_{k\in I_{i}^{2}\cap I_{j}^{2}}a_{k}^{\prime }\cdot \prod_{k\in I_{i}^{2}\cap \bar{I_{j}^{2}}}a_{k}\cdot \prod_{k\in \bar{I_{i}^{2}}\cap \bar{I_{j}^{2}}\cap \left\{m+1\right\}}b_{k}^{\prime }\cdot \prod_{k\in \bar{I_{i}^{2}}\cap I_{j}^{2}}b_{k},$$

where $\alpha_{ij}\left(m+1\right)$s are given by (19). This time, there are four cases to consider.

Suppose $i\leq 2^{m}$ and $j\leq 2^{m}$. Then, $I_{i}^{2}=\emptyset$, $I_{j}^{2}=\emptyset$, $\bar{I_{i}^{2}}=N_{m+1}$, $\bar{I_{j}^{2}}=N_{m+1}$ and

$$f_{1}\left(i\right)=\left(i-1\right)\;\mathrm{div}\;2^{m}+1=1,\;f_{2}\left(j\right)=\left(j-1\right)\;\mathrm{div}\;2^{m}+1=1,$$

so (27) takes the form

$$\alpha_{11}\left(m+1\right)=b_{m+1}^{\prime }=\prod_{k\in \{m+1\}}b_{k}^{\prime }.$$

If $i\leq 2^{m}$ and $j\in \{2^{m}+1,\ldots ,2^{m+1}\}$, then $I_{i}^{2}=\emptyset$, $I_{j}^{2}=\left\{m+1\right\}$, $\bar{I_{i}^{2}}=N_{m+1}$, $\bar{I_{j}^{2}}=N_{m}$, with $f_{1}\left(i\right)=1$ and $f_{2}\left(j\right)=2$. Thus, we have

$$\alpha_{12}\left(m+1\right)=b_{m+1}=\prod_{k\in \{m+1\}}b_{k}.$$

In a similar way, if $i\in \{2^{m}+1,\ldots ,2^{m+1}\}$ and $j\leq 2^{m}$, then $I_{i}^{2}=\left\{m+1\right\}$, $I_{j}^{2}=\emptyset$, $\bar{I_{i}^{2}}=N_{m}$, $\bar{I_{j}^{2}}=N_{m+1}$, with $f_{1}\left(i\right)=2$ and $f_{2}\left(j\right)=1$. Therefore, in this case

$$\alpha_{21}\left(m+1\right)=a_{m+1}=\prod_{k\in \{m+1\}}a_{k}.$$

Finally, for $i,j\in \{2^{m}+1,\ldots ,2^{m+1}\}$ we have $I_{i}^{2}=I_{j}^{2}=\left\{m+1\right\}$, $\bar{I_{i}^{2}}=\bar{I_{j}^{2}}=N_{m}$ and $f_{1}\left(i\right)=2$, $f_{2}\left(j\right)=2$. Hence, we obtain

$$\alpha_{22}\left(m+1\right)=a_{m+1}^{\prime }=\prod_{k\in \{m+1\}}a_{k}^{\prime }.$$

This proves (27), which implies (25). In consequence, (20) holds true and the proof of the theorem is completed. □

**Example 2.**  *Given Theorem 2 and* (14)*, the four eigenvalues from Example 1 can be expressed in the form*

$$\left\{\begin{array}{c} \lambda_{1}=\mu_{1}^{2}\mu_{1}^{1}=1\cdot 1,\\ \lambda_{2}=\mu_{1}^{2}\mu_{2}^{1}=1\cdot \left(a_{1}^{\prime }-b_{1}\right),\\ \lambda_{3}=\mu_{2}^{2}\mu_{1}^{1}=\left(a_{2}^{\prime }-b_{2}\right)\cdot 1,\\ \lambda_{4}=\mu_{2}^{2}\mu_{2}^{1}=\left(a_{2}^{\prime }-b_{2}\right)\cdot \left(a_{1}^{\prime }-b_{1}\right), \end{array}\right.$$

*and their corresponding eigenvectors as tensor products*

$$\left\{\begin{array}{c} x_{\lambda_{1}}=v_{1}^{2}⊗v_{1}^{1}=\left(1,\frac{b_{2}}{a_{2}}\right)⊗\left(1,\frac{b_{1}}{a_{1}}\right)=\left(1,\frac{b_{1}}{a_{1}},\frac{b_{2}}{a_{2}},\frac{b_{1}b_{2}}{a_{1}a_{2}}\right),\\ x_{\lambda_{2}}=v_{1}^{2}⊗v_{2}^{1}=\left(1,\frac{b_{2}}{a_{2}}\right)⊗\left(1,-1\right)=\left(1,-1,\frac{b_{2}}{a_{2}},-\frac{b_{2}}{a_{2}}\right),\\ x_{\lambda_{3}}=v_{2}^{2}⊗v_{1}^{1}=\left(1,-1\right)⊗\left(1,\frac{b_{1}}{a_{1}}\right)=\left(1,\frac{b_{1}}{a_{1}},-1,-\frac{b_{1}}{a_{1}}\right),\\ x_{\lambda_{4}}=v_{2}^{2}⊗v_{2}^{1}=\left(1,-1\right)⊗\left(1,-1\right)=\left(1,-1,-1,1\right). \end{array}\right.$$

*Using the tensor product notation, the stationary distribution π (see* (8)*) may be written as*

$$\pi =\frac{1}{\prod_{k=1}^{m}\left(1+\frac{b_{k}}{a_{k}}\right)}\cdot \left(1,\frac{b_{m}}{a_{m}}\right)⊗\left(1,\frac{b_{m-1}}{a_{m-1}}\right)⊗\ldots ⊗\left(1,\frac{b_{1}}{a_{1}}\right).$$

## 3. A Discrete Version of Blackwell’s Markov Chain

Let $X_{1}\left(\cdot \right),X_{2}\left(\cdot \right),X_{3}\left(\cdot \right),\ldots$ be an infinite sequence of mutually independent, two-state chains as described in Section 2. A discrete analogue of the Markov chain introduced in [1] can be defined as follows:

(28) $$X\left(n\right)≔\left(X_{1}\left(n\right),X_{2}\left(n\right),X_{3}\left(n\right),\ldots \right)\in {\left\{0,1\right\}}^{\infty },\;n\geq 0.$$

This joint process is well-defined by the Kolmogorov extension theorem (see [3] (p. 65)), and it is also a Markov process. At this point, however, it is not clear whether it is a Markov chain, i.e., that its state space is countable. We deal with this issue in Lemma 2.

Let us first notice that ${\left(X\left(n\right)\right)}_{n\geq 0}$ is characterized by two sequences ${\left(a_{k}\right)}_{k\in N}$ and ${\left(b_{k}\right)}_{k\in N}$, where $a_{k},b_{k}\in \left(0,1\right)$ for k≥1 (see (3)). As noted in the introduction, there is no notion of an instantaneous state for a discrete-time Markov chain.

In a similar way as in [6] (Lemma 5), we show that under certain conditions imposed on ${\left(a_{k}\right)}_{k\in N}$ and ${\left(b_{k}\right)}_{k\in N}$, the set of states of ${\left(X\left(n\right)\right)}_{n\geq 0}$ can be regarded as N.

**Lemma 2.**  *Let* S *denote the state space of the Markov process defined by* (28)*. If the sequence* ${\left(b_{k}\right)}_{k\in N}$ *satisfies*

(29) $$\sum_{k=1}^{\infty }b_{k}<+\infty ,$$

*then* S *is countable and it can be identified with* N*. The transition matrix* $P={\left(p_{ij}\right)}_{i,j\in N}$ *of this chain equals*

(30) $$p_{ij}=\prod_{k\in I_{i}\cap I_{j}}a_{k}^{\prime }\cdot \prod_{k\in I_{i}\cap \bar{I_{j}}}a_{k}\cdot \prod_{k\in \bar{I_{i}}\cap \bar{I_{j}}}b_{k}^{\prime }\cdot \prod_{k\in \bar{I_{i}}\cap I_{j}}b_{k}.$$

*Moreover, every* $p_{ij}\in \left(0,1\right)$ *and it is a product of infinitely many numbers.*

**Proof.**  For now, we have $S\subset {\left\{0,1\right\}}^{\infty }$. The set ${\left\{0,1\right\}}^{\infty }$, consisting of all sequences of 0 s and 1 s, including those with infinitely many 1 s, is uncountable by the known Cantor’s diagonal argument. So, in general, S could be uncountable.

However, if the condition (29) holds true, then, with probability one, elements of S are sequences with a finite number of 1 s. This follows from the Borel–Cantelli lemma (see, e.g., [3] (p. 66)). Namely, we denote $A_{k}≔\left\{X_{k}\left(n+1\right)=1|X_{k}\left(n\right)=0\right\}$, where k≥1. Then, $P\left(A_{k}\right)=b_{k}$ (see (3)) and $P(A_{k}\;i.o.)=0$, provided that

$$\sum_{k=1}^{\infty }P\left(A_{k}\right)=\sum_{k=1}^{\infty }b_{k}<+\infty .$$

In other words, the condition (29) guarantees that only a finite number of 0 s change to 1 s. Formally, we can write S as

$$S=\left\{{\left(\delta_{k}\right)}_{k\in N}:\delta_{k}=0\;\mathrm{or}\;1\;\mathrm{and}\;\;\sum_{k=1}^{\infty }\delta_{k}<+\infty \right\}$$

and the set thus defined is countable, as a countable sum of finite sets. Because S is infinite and countable, there exists a bijection between S and N. We define f:S→N by

(31) $$f\left(\left(\delta_{1},\delta_{2},\delta_{3},\ldots \right)\right)=1+\sum_{k=1}^{\infty }\delta_{k}2^{k-1}<+\infty$$

(see also (6)). That *f* is a bijection is a consequence of the fact that every natural number has a unique binary representation, which is encoded in ${\left(\delta_{k}\right)}_{k\in N}$. So we may write (31) in an equivalent way f(δ)=i, where

(32) $$S∋\delta =\left(\delta_{1},\delta_{2},\ldots ,\delta_{m},0,0,0,\ldots \right)\;⟷\;i=1+\sum_{k=1}^{m}\delta_{k}2^{k-1}\in N,$$

In contrast to (6), where *m* is fixed, an integer *m* in (32) is not fixed, but depends on δ. For every δ∈S such an *m* exists, because it has a finite number of 1 s. Therefore, S can be identified with N, which means

P(X(n)∈N|X(0)∈N)=1,for alln≥0.

From now on we assume S=N. Fix i,j∈N and let $I_{i}$, $I_{j}$ be their representation sets determined by (4). Let $\delta ≔f^{-1}\left(i\right)$ and $\delta^{\prime }≔f^{-1}\left(j\right)$, where *f* is defined by (31). This time, $I_{i}$ and $I_{j}$, are subsets of N with $\bar{I_{i}}=N\setminus I_{i}$ and $\bar{I_{j}}=N\setminus I_{j}$. Therefore, we have

(33) $$N=\underset{1^{\prime }s\to 1^{\prime }s}{\underset{︸}{\left(I_{i}\cap I_{j}\right)}}\cup \underset{1^{\prime }s\to 0^{\prime }s}{\underset{︸}{\left(I_{i}\cap \bar{I_{j}}\right)}}\cup \underset{0^{\prime }s\to 0^{\prime }s}{\underset{︸}{\left(\bar{I_{i}}\cap \bar{I_{j}}\right)}}\cup \underset{0^{\prime }s\to 1^{\prime }s}{\underset{︸}{\left(\bar{I_{i}}\cap I_{j}\right)}}.$$

This is an analogous situation to that of (7) and the meaning of the four pairwise disjoint sets in (33) is similar. What is different here is that the set $\bar{I_{i}}\cap \bar{I_{j}}$ is infinite. In other words, infinitely many 0 s change to 0 s. Notice that the sets $I_{i}\cap I_{j}$, $I_{i}\cap \bar{I_{j}}$ and $\bar{I_{i}}\cap I_{j}$ are finite. Therefore, to justify (30) we need to prove that the infinite product $\prod_{k\in \bar{I_{i}}\cap \bar{I_{j}}}b_{k}^{\prime }$ converges. It is enough to consider the case of $p_{11}$ and to prove

(34) $$p_{11}=\prod_{k=1}^{\infty }b_{k}^{\prime }=\prod_{k=1}^{\infty }\left(1-b_{k}\right)\in \left(0,1\right).$$

Because we assume $b_{k}\in \left(0,1\right)$, for every k≥1, it turns out the conditions (29) and (34) are equivalent (see [17] (Problem 3.8.4)). Thus, (29) implies (34). Moreover, (34) implies that $p_{ij}\in \left(0,1\right)$, for any i,j∈N, since the product defining $p_{ij}$ differs from $p_{11}$ by a finite number of terms. This completes the proof of (30). □

**Lemma 3.**  *The matrix given by* (30) *is stochastic; i.e., it satisfies*

(35) $$\sum_{j=1}^{\infty }p_{ij}=1,\;for\;every\;i\in N.$$

**Proof.**  For fixed i∈N we have

(36) $$\sum_{j=1}^{\infty }p_{ij}=\sum_{I_{j}\subset N}\left(\prod_{k\in I_{i}\cap I_{j}}a_{k}^{\prime }\cdot \prod_{k\in I_{i}\cap \bar{I_{j}}}a_{k}\cdot \prod_{k\in \bar{I_{i}}\cap \bar{I_{j}}}b_{k}^{\prime }\cdot \prod_{k\in \bar{I_{i}}\cap I_{j}}b_{k}\right),$$

where the summation runs over all finite subsets of N. Denote $I≔I_{j}\cap I_{i}$ and $I^{\prime }≔I_{j}\cap \bar{I_{i}}$. The sets $I,I^{\prime }$ are disjoint sets, $I\cap I^{\prime }=\emptyset$, with $I\subset I_{i}$ and $I^{\prime }\subset \bar{I_{i}}$. Therefore, we write (36) as

$$\sum_{j=1}^{\infty }p_{ij}=\sum_{I\subset I_{i}}\left[\sum_{I^{\prime }\subset \bar{I_{i}}}\left(\prod_{k\in I}a_{k}^{\prime }\cdot \prod_{k\in I_{i}\cap \bar{I}}a_{k}\cdot \prod_{k\in \bar{I_{i}}\cap \bar{I^{\prime }}}b_{k}^{\prime }\cdot \prod_{k\in I^{\prime }}b_{k}\right)\right],$$

where the outer sum is over all finite subsets of $I_{i}$ and the interior sum is over all finite subsets of $\bar{I_{i}}$ (see Section 4.2). Moreover, notice that in the interior sum, we can factor out the products that do not depend on *I*, namely,

$$\sum_{j=1}^{\infty }p_{ij}=\sum_{I\subset I_{i}}\left[\left(\prod_{k\in I}a_{k}^{\prime }\cdot \prod_{k\in I_{i}\cap \bar{I}}a_{k}\right)\cdot \sum_{I^{\prime }\subset \bar{I_{i}}}\left(\prod_{k\in \bar{I_{i}}\cap \bar{I^{\prime }}}b_{k}^{\prime }\cdot \prod_{k\in I^{\prime }}b_{k}\right)\right].$$

Based on Lemma 6, we have

$$\sum_{I^{\prime }\subset \bar{I_{i}}}\left(\prod_{k\in \bar{I_{i}}\cap \bar{I^{\prime }}}b_{k}^{\prime }\cdot \prod_{k\in I^{\prime }}b_{k}\right)=\prod_{k\in \bar{I_{i}}}\left(b_{k}+b_{k}^{\prime }\right)=1,$$

and using (83), we obtain

$$\sum_{j=1}^{\infty }p_{ij}=\sum_{I\subset I_{i}}\left(\prod_{k\in I}a_{k}^{\prime }\cdot \prod_{k\in I_{i}\cap \bar{I}}a_{k}\right)=\prod_{k\in I_{i}}\left(a_{k}+a_{k}^{\prime }\right)=1,$$

which proves (35). □

The (infinite) matrix $P={\left(p_{ij}\right)}_{i,j\in N}$, as a Markov operator, acts in $l^{1}\left(N\right)$. Recall this is the Banach space of all absolutely summable sequences, which means that $x={\left(\xi_{i}\right)}_{i\in N}$ belongs to $l^{1}\left(N\right)$ if and only if $\sum_{i\in N}\left|\xi_{i}\right|<+\infty$, and then ${∥x∥}_{l^{1}\left(N\right)}=\sum_{i\in N}\left|\xi_{i}\right|$. It is a separable space in which every $x\in l^{1}\left(N\right)$ can be written as $\sum_{i\in N}\xi_{i}e_{i}$, where ${\left\{e_{i}\right\}}_{i\in N}$ is the standard Schauder basis in this space, i.e., $e_{i}=\left(\ldots ,0,1,0,\ldots \right)$ with 1 in the *i*-th coordinate (see, e.g., [18] (Chapter 7)). In particular, ${∥x∥}_{l^{1}\left(N\right)}=∥x\cdot {P∥}_{l^{1}\left(N\right)}$ for any $x\in l^{1}\left(N\right)$ such that x≥0. If *x* is arbitrary, then $∥x\cdot {P∥}_{l^{1}\left(N\right)}\leq {∥x∥}_{l^{1}\left(N\right)}$, which means *P* is a linear contraction (see [2] (p. 15)). This lemma is an analogue of [6] (Theorem 2).

**Lemma 4.**  *Suppose the sequences* ${\left(a_{k}\right)}_{k\in N}$*,* ${\left(b_{k}\right)}_{k\in N}$ *satisfy* (29)*, and additionally*

(37) $$\sum_{k=1}^{\infty }\frac{b_{k}}{a_{k}}<+\infty .$$

*Then the stationary distribution* $\pi ={\left(\pi_{i}\right)}_{i\in N}$ *for the Markov chain defined by* (28) *equals*

(38) $$\pi_{i}=\frac{1}{\prod_{k=1}^{\infty }\left(1+\frac{b_{k}}{a_{k}}\right)}\cdot \prod_{k\in I_{i}}\frac{b_{k}}{a_{k}},$$

*and it satisfies the following detailed balance condition:*

(39) $$\pi_{i}p_{ij}=\pi_{j}p_{ji},\;i,j\in N.$$

**Proof.**  First, we prove $\sum_{i=1}^{\infty }\pi_{i}=1$, which means $\pi \in l^{1}\left(N\right)$. The condition (37) implies that the product $\prod_{k=1}^{\infty }\left(1+\frac{b_{k}}{a_{k}}\right)$ converges, and it can be written as

$$\prod_{k=1}^{\infty }\left(1+\frac{b_{k}}{a_{k}}\right)=1+\sum_{k=1}^{\infty }\frac{b_{k}}{a_{k}}+\sum_{k_{1}<k_{2}}\frac{b_{k_{1}}b_{k_{2}}}{a_{k_{1}}a_{k_{2}}}+\sum_{k_{1}<k_{2}<k_{3}}\frac{b_{k_{1}}b_{k_{2}}b_{k_{3}}}{a_{k_{1}}a_{k_{2}}a_{k_{3}}}+\ldots ,$$

or, equivalently using our notation, in the form

$$\prod_{k=1}^{\infty }\left(1+\frac{b_{k}}{a_{k}}\right)=\sum_{i=1}^{+\infty }\left(\prod_{k\in I_{i}}\frac{b_{k}}{a_{k}}\right)$$

See [17] (Problems 3.8.3, 3.8.26) and Section 4.1. This shows π is a probability distribution. Now we prove π is stationary. For this purpose, define $\sigma ={\left(\sigma_{i}\right)}_{i\in N}$, a multiple of π, as

(40) $$\sigma_{i}≔\pi_{i}\cdot \prod_{k=1}^{\infty }\left(1+\frac{b_{k}}{a_{k}}\right)=\prod_{k\in I_{i}}\frac{b_{k}}{a_{k}}.$$

We will prove that σ=σP and $\sigma_{i}p_{ij}=\sigma_{j}p_{ji}$, which is equivalent to π=πP and (39), respectively. We begin with $\sigma_{i}p_{ij}$. Based on (30) and (40), it equals

(41) $$\sigma_{i}p_{ij}=\prod_{k\in I_{i}}\frac{b_{k}}{a_{k}}\cdot \prod_{k\in I_{i}\cap I_{j}}a_{k}^{\prime }\cdot \prod_{k\in I_{i}\cap \bar{I_{j}}}a_{k}\cdot \prod_{k\in \bar{I_{i}}\cap \bar{I_{j}}}b_{k}^{\prime }\cdot \prod_{k\in \bar{I_{i}}\cap I_{j}}b_{k}.$$

Notice that $I_{i}\cup \left(\bar{I_{i}}\cap I_{j}\right)=I_{i}\cup I_{j}$; hence, we have

$$\prod_{k\in I_{i}}b_{k}\cdot \prod_{k\in \bar{I_{i}}\cap I_{j}}b_{k}=\prod_{k\in I_{i}\cup I_{j}}b_{k}=\prod_{k\in I_{j}}b_{k}\cdot \prod_{k\in \bar{I_{j}}\cap I_{i}}b_{k}$$

and

$$\prod_{k\in I_{i}}\frac{1}{a_{k}}\cdot \prod_{k\in I_{i}\cap \bar{I_{j}}}a_{k}=\prod_{k\in I_{i}\cap I_{j}}\frac{1}{a_{k}}=\prod_{k\in I_{j}}\frac{1}{a_{k}}\cdot \prod_{k\in I_{j}\cap \bar{I_{i}}}a_{k}.$$

Thus, we can write (41) as follows:

(42) $$\sigma_{i}p_{ij}=\prod_{k\in I_{j}}\frac{b_{k}}{a_{k}}\cdot \prod_{k\in I_{i}\cap I_{j}}a_{k}^{\prime }\cdot \prod_{k\in I_{j}\cap \bar{I_{i}}}a_{k}\cdot \prod_{k\in \bar{I_{i}}\cap \bar{I_{j}}}b_{k}^{\prime }\cdot \prod_{k\in \bar{I_{j}}\cap I_{i}}b_{k}=\sigma_{j}p_{ji}.$$

This proves (39). Now we prove σ=σP. Taking into account (42) and (35), we obtain

$${\left(\sigma P\right)}_{j}=\sum_{i\in N}\sigma_{i}p_{ij}=\sum_{i\in N}\sigma_{j}p_{ji}=\sigma_{j}\sum_{i\in N}p_{ji}=\sigma_{j},\;j\in N,$$

which proves π=πP. The above calculation shows the detailed balance condition is stronger than stationarity. In other words, not every stationary distribution must satisfy (39). This completes the proof. □

**Theorem 3.**  *Suppose the sequences* ${\left(a_{k}\right)}_{k\in N}$*,* ${\left(b_{k}\right)}_{k\in N}$ *satisfy* (29) *and* (37)*. Then the transition matrix* $P={\left(p_{ij}\right)}_{i,j\in N}$*, determined by* (30)*, has infinitely many eigenvalues given by*

(43) $$\lambda_{i}=\prod_{k\in I_{i}}\left(1-a_{k}-b_{k}\right)=\prod_{k\in I_{i}}\left(a_{k}^{\prime }-b_{k}\right),\;i\in N.$$

*The eigenvector* $x_{\lambda_{i}}=\left(\xi_{i,j}\right)$*, corresponding to* $\lambda_{i}$*, has the form*

(44) $$\xi_{i,j}={\left(-1\right)}^{|I_{j}\cap I_{i}|}\prod_{k\in I_{j}\cap \bar{I_{i}}}\frac{b_{k}}{a_{k}},\;j=1,2,3,\ldots .$$

*and its* $l^{1}$*-norm equals*

(45) $${∥x_{\lambda_{i}}∥}_{l^{1}\left(N\right)}=2^{|I_{i}|}\cdot \prod_{k\in \bar{I_{i}}}\left(1+\frac{b_{k}}{a_{k}}\right).$$

**Proof.**  Fix i∈N and let $y={\left(y_{j}\right)}_{j\in N}$ denote the product of $x_{\lambda_{i}}$ and *P*, i.e., $y=x_{\lambda_{i}}\cdot P$. We will show that

(46) $$y_{j}=\lambda_{i}\xi_{i,j},\;j\in N.$$

Now also fix j∈N. Then $y_{j}={\left(x_{\lambda_{i}}\cdot P\right)}_{j}$ and

$$y_{j}=\sum_{l=1}^{\infty }\xi_{i,l}\cdot p_{l,j}=\sum_{l=1}^{\infty }\left({\left(-1\right)}^{|I_{i}\cap I_{l}|}\prod_{k\in I_{l}\cap \bar{I_{i}}}\frac{b_{k}}{a_{k}}\prod_{k\in I_{l}\cap I_{j}}a_{k}^{\prime }\cdot \prod_{k\in I_{l}\cap \bar{I_{j}}}a_{k}\cdot \prod_{k\in \bar{I_{l}}\cap \bar{I_{j}}}b_{k}^{\prime }\cdot \prod_{k\in \bar{I_{l}}\cap I_{j}}b_{k}\right).$$

Equivalently, we may write $y_{j}$ as

(47) $$y_{j}=\sum_{I_{l}\subset N}\left({\left(-1\right)}^{|I_{i}\cap I_{l}|}\prod_{k\in I_{l}\cap \bar{I_{i}}}\frac{b_{k}}{a_{k}}\prod_{k\in I_{l}\cap I_{j}}a_{k}^{\prime }\cdot \prod_{k\in I_{l}\cap \bar{I_{j}}}a_{k}\cdot \prod_{k\in \bar{I_{l}}\cap \bar{I_{j}}}b_{k}^{\prime }\cdot \prod_{k\in \bar{I_{l}}\cap I_{j}}b_{k}\right),$$

where the sum is over all finite subsets of N. Because $I_{i}$ and $I_{j}$ are fixed, $I_{l}$ can be written as a sum of the following three pairwise disjoint sets

(48) $$I_{l}=\underset{=I}{\underset{︸}{\left(I_{l}\cap I_{i}\right)}}\cup \underset{=I^{\prime }}{\underset{︸}{\left(I_{l}\cap \bar{I_{i}}\cap I_{j}\right)}}\cup \underset{=I^{\prime \prime }}{\underset{︸}{\left(I_{l}\cap \bar{I_{i}}\cap \bar{I_{j}}\right)}}.$$

In particular, $I\subset I_{i}$, $I^{\prime }\subset \bar{I_{i}}\cap I_{j}$ and $I^{\prime \prime }\subset \bar{I_{i}}\cap \bar{I_{j}}$. Thanks to (48), equality (47) is equivalent to

(49) $$y_{j}=\sum_{I\subset I_{i}}\left[\sum_{I^{\prime }\subset I_{j}\cap \bar{I_{i}}}\left(\sum_{I^{\prime \prime }\subset \bar{I_{i}}\cap \bar{I_{j}}}{\left(-1\right)}^{\left|I\right|}\prod_{k\in J_{1}}\frac{b_{k}}{a_{k}}\prod_{k\in J_{2}}a_{k}^{\prime }\prod_{k\in J_{3}}a_{k}\prod_{k\in J_{4}}b_{k}^{\prime }\prod_{k\in J_{5}}b_{k}\right)\right],$$

where the first sum is over all subsets of $I_{i}$, the second sum is over all subsets of $I_{j}\cap \bar{I_{i}}$ and the third sum is over all finite subsets of $\bar{I_{i}}\cap \bar{I_{j}}$. The sets $J_{1},\ldots ,J_{5}$ in (49) are expressed in terms of *I*, $I^{\prime }$ and $I^{\prime \prime }$, and are given by

$$J_{1}=I^{\prime }\cup I^{\prime \prime },\;J_{2}=I^{\prime }\cup \left(I\cap I_{j}\right),\;J_{3}=I^{\prime \prime }\cup \left(I\cap \bar{I_{j}}\right),\;J_{4}=\bar{I_{j}}\cap \bar{I^{\prime \prime }}\cap \bar{I},\;J_{5}=I_{j}\cap \bar{I}\cap \bar{I^{\prime }}.$$

Of these five sets, $J_{4}$ is the only infinite one. To perform the summation over $I^{\prime \prime }$ in (49), first notice that $J_{4}$ can be written as a sum of two disjoint sets, namely,

$$J_{4}=\bar{I_{j}}\cap \bar{I^{\prime \prime }}\cap \bar{I}=\left(\bar{I_{j}}\cap \bar{I}\cap I_{i}\right)\cup \left(\bar{I_{j}}\cap \bar{I^{\prime \prime }}\cap \bar{I_{i}}\right).$$

This holds true because $I_{i}\cup \bar{I_{i}}=N$ and $I^{\prime \prime }$ is a subset of $\bar{I_{i}}\cap \bar{I_{j}}$. By Lemma 6, we have

$$\begin{array}{cc} \; & \sum_{I^{\prime \prime }\subset \bar{I_{i}}\cap \bar{I_{j}}}\left(\prod_{k\in I^{\prime \prime }}\frac{b_{k}}{a_{k}}\prod_{k\in I^{\prime \prime }}a_{k}\prod_{k\in \bar{I_{j}}\cap \bar{I^{\prime \prime }}\cap \bar{I_{i}}}b_{k}^{\prime }\right)\\ \; & =\sum_{I^{\prime \prime }\subset \bar{I_{i}}\cap \bar{I_{j}}}\left(\prod_{k\in I^{\prime \prime }}b_{k}\prod_{k\in \bar{I_{j}}\cap \bar{I^{\prime \prime }}\cap \bar{I_{i}}}b_{k}^{\prime }\right)=\prod_{k\in \bar{I_{i}}\cap \bar{I_{j}}}\left(b_{k}+b_{k}^{\prime }\right)=1, \end{array}$$

and (49) takes the form

$$\begin{array}{cc} y_{j} & =\sum_{I\subset I_{i}}\left[\sum_{I^{\prime }\subset I_{j}\cap \bar{I_{i}}}\left({\left(-1\right)}^{\left|I\right|}\prod_{k\in I^{\prime }}\frac{b_{k}}{a_{k}}\prod_{k\in J_{2}}a_{k}^{\prime }\prod_{k\in I\cap \bar{I_{j}}}a_{k}\prod_{k\in I_{i}\cap \bar{I}\cap \bar{I_{j}}}b_{k}^{\prime }\prod_{k\in J_{5}}b_{k}\right)\right]\\ \; & =\prod_{k\in I_{j}\cap \bar{I_{i}}}b_{k}\sum_{I\subset I_{i}}\left[\sum_{I^{\prime }\subset I_{j}\cap \bar{I_{i}}}\left({\left(-1\right)}^{\left|I\right|}\prod_{k\in \bar{I}\cap I_{i}\cap I_{j}}b_{k}\prod_{k\in I\cap \bar{I_{j}}}a_{k}\prod_{k\in I_{i}\cap \bar{I}\cap \bar{I_{j}}}b_{k}^{\prime }\prod_{k\in I\cap I_{j}}a_{k}^{\prime }\prod_{k\in I^{\prime }}\frac{a_{k}^{\prime }}{a_{k}}\right)\right]. \end{array}$$

The application of (83), together with $a_{k}+a_{k}^{\prime }=1$, gives

$$\sum_{I^{\prime }\subset I_{j}\cap \bar{I_{i}}}\left(\prod_{k\in I^{\prime }}\frac{a_{k}^{\prime }}{a_{k}}\right)=\prod_{k\in I_{j}\cap \bar{I_{i}}}\left(1+\frac{a_{k}^{\prime }}{a_{k}}\right)=\prod_{k\in I_{j}\cap \bar{I_{i}}}\frac{1}{a_{k}}.$$

This leads to

(50) $$y_{j}=\left(\prod_{k\in I_{j}\cap \bar{I_{i}}}\frac{b_{k}}{a_{k}}\right)\sum_{I\subset I_{i}}\left({\left(-1\right)}^{\left|I\right|}\prod_{k\in \bar{I}\cap I_{i}\cap I_{j}}b_{k}\prod_{k\in I\cap \bar{I_{j}}}a_{k}\prod_{k\in I_{i}\cap \bar{I}\cap \bar{I_{j}}}b_{k}^{\prime }\prod_{k\in I\cap I_{j}}a_{k}^{\prime }\right).$$

In view of (43), (44) and (50), to prove (46), we need to show

(51) $$\lambda_{i}={\left(-1\right)}^{|I_{j}\cap I_{i}|}\sum_{I\subset I_{i}}\left({\left(-1\right)}^{\left|I\right|}\prod_{k\in \bar{I}\cap I_{i}\cap I_{j}}b_{k}\prod_{k\in I\cap \bar{I_{j}}}a_{k}\prod_{k\in I_{i}\cap \bar{I}\cap \bar{I_{j}}}b_{k}^{\prime }\prod_{k\in I\cap I_{j}}a_{k}^{\prime }\right).$$

So we prove that right hand sides of (43) and (51) are equal. We begin with (43). First, from $I_{i}=\left(I_{i}\cap I_{j}\right)\cup \left(I_{i}\cap \bar{I_{j}}\right)$ we have

$$\lambda_{i}=\prod_{k\in I_{i}}\left(a_{k}^{\prime }-b_{k}\right)=\prod_{k\in I_{i}\cap I_{j}}\left(a_{k}^{\prime }-b_{k}\right)\prod_{k\in I_{i}\cap \bar{I_{j}}}\left(a_{k}^{\prime }-b_{k}\right).$$

The set $I_{i}\cap I_{j}$ is finite, so using (84), we can write

(52) $$\prod_{k\in I_{i}\cap I_{j}}\left(a_{k}^{\prime }-b_{k}\right)=\sum_{I^{\prime }\subset I_{i}\cap I_{j}}\left({\left(-1\right)}^{|\bar{I^{\prime }}\cap I_{i}\cap I_{j}|}\prod_{k\in I^{\prime }}a_{k}^{\prime }\prod_{k\in \bar{I^{\prime }}\cap I_{i}\cap I_{j}}b_{k}\right).$$

Because $a_{k}^{\prime }-b_{k}=-\left(a_{k}-b_{k}^{\prime }\right)$, then

$$\prod_{k\in I_{i}\cap \bar{I_{j}}}\left(a_{k}^{\prime }-b_{k}\right)={\left(-1\right)}^{|I_{i}\cap \bar{I_{j}}|}\prod_{k\in I_{i}\cap \bar{I_{j}}}\left(a_{k}-b_{k}^{\prime }\right)$$

and from this, again using (84), we have

(53) $$\prod_{k\in I_{i}\cap \bar{I_{j}}}\left(a_{k}^{\prime }-b_{k}\right)={\left(-1\right)}^{|I_{i}\cap \bar{I_{j}}|}\sum_{I^{\prime \prime }\subset I_{i}\cap \bar{I_{j}}}\left({\left(-1\right)}^{|\bar{I^{\prime \prime }}\cap I_{i}\cap \bar{I_{j}}|}\prod_{k\in I^{\prime \prime }}a_{k}\prod_{k\in \bar{I^{\prime \prime }}\cap I_{i}\cap \bar{I_{j}}}b_{k}^{\prime }\right).$$

To combine (52) and (53), notice that the sets $I^{\prime }$ and $I^{\prime \prime }$ in (52) and (53), respectively, are disjoint sets and are subsets of $I_{i}$. Then, the set $I≔I^{\prime }\cup I^{\prime \prime }$ is also a subset of $I_{i}$ with $I^{\prime }=I\cap I_{j}$, $I^{\prime \prime }=I\cap \bar{I_{j}}$. Moreover,

$$|\bar{I^{\prime }}\cap I_{i}\cap I_{j}\left|+\right|\bar{I^{\prime \prime }}\cap I_{i}\cap \bar{I_{j}}\left|=\right|\bar{I}\cap I_{i}|$$

and

$${\left(-1\right)}^{|\bar{I}\cap I_{i}|}={\left(-1\right)}^{|I_{i}\left|-|I\right|}={\left(-1\right)}^{|I_{i}|}\cdot {\left(-1\right)}^{\left|I\right|}.$$

Combining (52) and (53) results in

(54) $$\lambda_{i}={\left(-1\right)}^{|I_{i}\cap \bar{I_{j}}\left|+\right|I_{i}|}\sum_{I\subset I_{i}}\left({\left(-1\right)}^{\left|I\right|}\prod_{k\in \bar{I}\cap I_{i}\cap I_{j}}b_{k}\prod_{k\in I\cap \bar{I_{j}}}a_{k}\prod_{k\in \bar{I}\cap I_{i}\cap \bar{I_{j}}}b_{k}^{\prime }\prod_{k\in I\cap I_{j}}a_{k}^{\prime }\right).$$

The final step is to notice

$$|I_{i}\cap \bar{I_{j}}\left|+\right|I_{i}\left|=2\right|I_{i}\cap \bar{I_{j}}\left|+\right|I_{i}\cap I_{j}|$$

and

$${\left(-1\right)}^{|I_{i}\cap \bar{I_{j}}\left|+\right|I_{i}|}={\left(-1\right)}^{|I_{i}\cap I_{j}|}.$$

This concludes the proof of (51); i.e., the right hand sides of (51) and (54) are equal.

Now we prove (45). Based on (44), we have

(55) $${∥x_{\lambda_{i}}∥}_{l^{1}\left(N\right)}=\sum_{j=1}^{\infty }\left(\prod_{k\in I_{j}\cap \bar{I_{i}}}\frac{b_{k}}{a_{k}}\right)=\sum_{I_{j}\subset N}\left(\prod_{k\in I_{j}\cap \bar{I_{i}}}\frac{b_{k}}{a_{k}}\right),$$

where the sum is over all finite subsets of N. Denote $I≔I_{j}\cap I_{i}$ and $I^{\prime }≔I_{j}\cap \bar{I_{i}}$. Then, $I\cap I^{\prime }=\emptyset$ and (55) becomes

$${∥x_{\lambda_{i}}∥}_{l^{1}\left(N\right)}=\sum_{I\subset I_{i}}\left[\sum_{I^{\prime }\subset \bar{I_{i}}}\left(\prod_{k\in I^{\prime }}\frac{b_{k}}{a_{k}}\right)\right],$$

where the outer sum runs over all subsets of $I_{i}$ and the interior sum is over all finite subsets of $\bar{I_{i}}=N\setminus I_{i}$. From (37) and Lemma 6, we have

$$\sum_{I^{\prime }\subset \bar{I_{i}}}\left(\prod_{k\in I^{\prime }}\frac{b_{k}}{a_{k}}\right)=\prod_{k\in \bar{I_{i}}}\left(1+\frac{b_{k}}{a_{k}}\right)<+\infty .$$

The set $I_{i}$ is finite and the number of all its subsets equals $2^{|I_{i}|}$; therefore,

$$∥x_{\lambda_{i}}{∥}_{l^{1}\left(N\right)}=\prod_{k\in \bar{I_{i}}}\left(1+\frac{b_{k}}{a_{k}}\right)\cdot \sum_{I\subset I_{i}}1=2^{|I_{i}|}\cdot \prod_{k\in \bar{I_{i}}}\left(1+\frac{b_{k}}{a_{k}}\right).$$

This completes the proof of (45) and the theorem. □

**Remark 2.**  *As in the finite case, we have* $\lambda_{1}=1$ *and* $|\lambda_{i}|<1$*, for* i≥2*. Although there are infinitely many eigenvalues, not necessarily distinct, each is expressed as a finite product. All vectors given by* (44) *are linearly independent (see Lemma 5). Moreover, it should be added the conditions* (29) *and* (37) *are not contradictory. For example, the sequences*

$$b_{k}=\frac{1}{3^{k}},\;a_{k}=\frac{1}{2^{k}},\;k\geq 1,$$

*satisfy both* (29) *and* (37)*.*

The results in Theorem 3 are formulated from the point of view of the Banach space $l^{1}\left(N\right)$ and the map x→x·P. These results can also be looked at from a slightly better and more natural perspective. Namely, that of the Hilbert space $\ell^{2}\left(\pi \right)$. This is shown in Theorem 4. To make everything precise, we start by recalling a few concepts.

The Banach space of all bounded sequences, usually denoted as $l^{\infty }\left(N\right)$, is a vector space equipped with the norm ${∥x∥}_{l^{\infty }\left(N\right)}≔\sup_{i\in N}\left|\xi_{i}\right|$. Thus, a sequence $x={\left(\xi_{i}\right)}_{i\in N}$ belongs to $l^{\infty }\left(N\right)$ if and only if $\sup_{i\in N}\left|\xi_{i}\right|<+\infty$. Let $P^{T}$ denote the transpose of *P* given by (30). The transition operator *T*, associated with *P*, is the map defined by

(56) $$Tx≔x\cdot P^{T},\;x\in l^{\infty }\left(N\right)$$

(see [19] (p. 113)). For this definition to be correct, we assume (as before) that *x* is a row vector. We can also write *T* in coordinates. To this end, let ${\left(\zeta_{i}\right)}_{i\in N}≔Tx$. Then, $\zeta_{j}=\sum_{i\in N}\xi_{i}p_{ji}$ for j∈N. It is clear that *T* is a linear operator. Moreover, one can show that ${∥Tx∥}_{l^{\infty }\left(N\right)}\leq {∥x∥}_{l^{\infty }\left(N\right)}$, which means it is a contraction.

Let π be the probability distribution given by (38). We define $\ell^{2}\left(\pi \right)$ as the Hilbert space of all infinite sequences of complex numbers equipped with the inner product

$${\langle x,y\rangle }_{\pi }≔\sum_{i=1}^{\infty }\xi_{i}\bar{\eta_{i}}\pi_{i},\;x={\left(\xi_{i}\right)}_{i\in N},\;y={\left(\eta_{i}\right)}_{i\in N},$$

and the corresponding norm ${∥x∥}_{\pi }=(\sum_{i=1}^{\infty }|\xi_{i}{{|}^{2}\pi_{i})}^{1/2}$. In other words, $x\in \ell^{2}\left(\pi \right)$ if and only if $\sum_{i=1}^{\infty }{\left|\xi_{i}\right|}^{2}\pi_{i}<+\infty$. For example, a constant vector (1,1,1,…) is an element of $\ell^{2}\left(\pi \right)$, because

$${∥(1,1,1,\ldots )∥}_{\pi }^{2}=\sum_{i=1}^{\infty }\pi_{i}=1<+\infty .$$

In a similar way, it can be shown that any $x\in l^{\infty }\left(N\right)$ belongs to $\ell^{2}\left(\pi \right)$, i.e., $l^{\infty }\left(N\right)\subset \ell^{2}\left(\pi \right)$ (see [19] (p. 119)). So for now, the domain of *T* is $l^{\infty }\left(N\right)$.

**Theorem 4.**  *Let P and π be given by* (30) *and* (38)*, respectively. Then, the transition operator T, defined by* (56)*, can be extended to* $\ell^{2}\left(\pi \right)$*. Such an extended operator is contractive,* ${∥Tx∥}_{\pi }\leq {∥x∥}_{\pi }$*, and self-adjoint in* $\ell^{2}\left(\pi \right)$*; i.e., it satisifies*

$${\langle Tx,y\rangle }_{\pi }={\langle x,Ty\rangle }_{\pi },\;x,y\in \ell^{2}\left(\pi \right).$$

*As a result, the spectrum of T is real and its point spectrum* $\sigma_{p}\left(T\right)$*, which is defined as the set of eigenvalues of T, equals*

(57) $$\sigma_{p}\left(T\right)=\sigma_{p}\left(P^{T}\right)=\sigma_{p}\left(P\right)=\left\{\lambda_{i}:i\in N\right\},$$

*where* $\lambda_{i}$ *is given by* (43)*. The eigenvector* $y_{\lambda_{i}}=\left(\eta_{j,i}\right)$*, corresponding to* $\lambda_{i}$*, has the form*

(58) $$\eta_{j,i}={\left(-1\right)}^{|I_{j}\cap I_{i}|}\prod_{k\in I_{i}\cap \bar{I_{j}}}\frac{b_{k}}{a_{k}},\;j=1,2,3,\ldots .$$

*Moreover, the eigenvectors* $y_{\lambda_{1}},y_{\lambda_{2}},\ldots$ *form an orthogonal family in* $\ell^{2}\left(\pi \right)$ *and*

(59) $${\langle y_{\lambda_{i_{1}}},y_{\lambda_{i_{2}}}\rangle }_{\pi }=\left\{\begin{array}{cc} ∥y_{\lambda_{i_{1}}}{∥}_{\pi }^{2}=\prod_{k\in I_{i_{1}}}\frac{b_{k}}{a_{k}}, & \mathrm{if}\;i_{1}=i_{2},\\ 0, & \mathrm{if}\;i_{1}\neq i_{2}. \end{array}\right.$$

**Proof.**  *P* and π satisfy (39), so *T* is a self-adjoint operator (see, e.g., [19] (pp. 119–120)). It is well known that the spectrum of a self-adjoint operator is real (see [18] (p. 301) and [18] (p. 309) for the proof of (57)). The argument that $y_{\lambda_{i}}\cdot P^{T}=\lambda_{i}y_{\lambda_{i}}$ is as follows. First, Theorem 3 says that $x_{\lambda_{i}}\cdot P=\lambda_{i}x_{\lambda_{i}}$. Equivalently, it means WP=DW, where the matrices *D* and *W* are given by (64) and (65), respectively. In Lemma 5, we prove that $W^{-1}=c^{-1}W$ for some c>0. Thus, $W=cW^{-1}$. As a result, $P=W^{-1}DW$ and $P^{T}=W^{T}D{\left(W^{-1}\right)}^{T}$. Finally, we obtain

$$P^{T}={\left(cW^{-1}\right)}^{T}D{\left(c^{-1}W\right)}^{T}={\left(W^{T}\right)}^{-1}DW^{T}.$$

The above equality shows that the rows of $W^{T}$, or equivalently, the columns of *W*, are eigenvectors of $P^{T}$ corresponding to its eigenvalues. But that is how $y_{\lambda_{i}}$’s are defined; i.e., $y_{\lambda_{i}}$ determined by (58) is the *i*-th column of *W*. This proves $y_{\lambda_{i}}P^{T}=\lambda_{i}y_{\lambda_{i}}$.

What remains to be proven is (59). To carry out the proof, let us denote

$$c_{k}≔\frac{b_{k}}{a_{k}},\;c≔\prod_{k=1}^{\infty }\left(1+\frac{b_{k}}{a_{k}}\right)$$

(see also Section 4). With this notation, we have

(60) $$∥y_{\lambda_{i_{1}}}{∥}_{\pi }^{2}=\sum_{j=1}^{\infty }\eta_{j,i_{1}}^{2}\pi_{j}=\frac{1}{c}\sum_{j=1}^{\infty }\left(\prod_{k\in I_{i_{1}}\cap \bar{I_{j}}}c_{k}^{2}\cdot \prod_{k\in I_{j}}c_{k}\right).$$

It is clear that the set $I_{i_{1}}\cup I_{j}$ equals $I_{i_{1}}\cup \left(I_{j}\cap \bar{I_{i_{1}}}\right)$. Then, in (60), we can factor out the product $\prod_{k\in I_{i_{1}}}c_{k}$, which gives

(61) $$∥y_{\lambda_{i_{1}}}{∥}_{\pi }^{2}=\frac{1}{c}\prod_{k\in I_{i_{1}}}c_{k}\cdot \sum_{j=1}^{\infty }\left(\prod_{k\in I_{i_{1}}\cap \bar{I_{j}}}c_{k}\cdot \prod_{k\in I_{j}\cap \bar{I_{i_{1}}}}c_{k}\right)=\prod_{k\in I_{i_{1}}}c_{k}$$

(see (75)), where the key argument used to prove it is the set $\left(I_{i_{1}}\cap \bar{I_{j}}\right)\cup \left(I_{j}\cap \bar{I_{i_{1}}}\right)$ equals the symmetric difference of $I_{i_{1}}$ and $I_{j}$ (see Section 4.1). Now let $i_{1}\neq i_{2}$. Then

(62) $${\langle y_{\lambda_{i_{1}}},y_{\lambda_{i_{2}}}\rangle }_{\pi }=\frac{1}{c}\sum_{j=1}^{\infty }\left[\left({\left(-1\right)}^{|I_{i_{1}}\cap I_{j}|}\prod_{k\in I_{i_{1}}\cap \bar{I_{j}}}c_{k}\right)\left({\left(-1\right)}^{|I_{i_{2}}\cap I_{j}|}\prod_{k\in I_{i_{2}}\cap \bar{I_{j}}}c_{k}\right)\left(\prod_{k\in I_{j}}c_{k}\right)\right].$$

Notice that

$$\left(I_{i_{1}}\cap \bar{I_{j}}\right)\cap \left(I_{i_{2}}\cap \bar{I_{j}}\right)=\left(I_{i_{1}}\cap I_{i_{2}}\right)\cap \bar{I_{j}},$$

so Equation (62) takes the form

$${\langle y_{\lambda_{i_{1}}},y_{\lambda_{i_{2}}}\rangle }_{\pi }=\frac{1}{c}\prod_{k\in I_{i_{1}}}c_{k}\cdot \sum_{j=1}^{\infty }\left[\left({\left(-1\right)}^{|I_{j}\cap I_{i_{1}}|}\prod_{k\in I_{j}\cap \bar{I_{i_{1}}}}c_{k}\right)\left({\left(-1\right)}^{|I_{j}\cap I_{i_{2}}|}\prod_{k\in \bar{I_{j}}\cap I_{i_{2}}}c_{k}\right)\right]=0,$$

see (77) and (82). This concludes the proof of (59). □

**Example 3.**  *As noted in Remark 1, the Formula* (12) *is also true when D and W are infinite-dimensional matrices. In Remark 3, we give a more detailed explanation of the right hand side of the formula*

(63) $$P^{n}=\frac{1}{\prod_{k=1}^{\infty }\left(1+\frac{b_{k}}{a_{k}}\right)}WD^{n}W,\;n\geq 1,$$

*where the diagonal matrix D equals*

(64) $$D=\left[\begin{array}{cccccc} 1 & 0 & 0 & 0 & 0 & \ldots \\ 0 & a_{1}^{\prime }-b_{1} & 0 & 0 & 0 & \ldots \\ 0 & 0 & a_{2}^{\prime }-b_{2} & 0 & 0 & \ldots \\ 0 & 0 & 0 & \left(a_{1}^{\prime }-b_{1}\right)\left(a_{2}^{\prime }-b_{2}\right) & 0 & \ldots \\ 0 & 0 & 0 & 0 & a_{3}^{\prime }-b_{3} & \ldots \\ \ldots & \ldots & \ldots & \ldots & \ldots & \ldots \end{array}\right]$$

*and*

(65) $$W=\left[\xi_{i,j}\right]=\left[\begin{array}{cccccccccc} 1 & \frac{b_{1}}{a_{1}} & \frac{b_{2}}{a_{2}} & \frac{b_{1}b_{2}}{a_{1}a_{2}} & \frac{b_{3}}{a_{3}} & \frac{b_{1}b_{3}}{a_{1}a_{3}} & \frac{b_{2}b_{3}}{a_{2}a_{3}} & \frac{b_{1}b_{2}b_{3}}{a_{1}a_{2}a_{3}} & \frac{b_{4}}{a_{4}} & \ldots \\ 1 & -1 & \frac{b_{2}}{a_{2}} & -\frac{b_{2}}{a_{2}} & \frac{b_{3}}{a_{3}} & -\frac{b_{3}}{a_{3}} & \frac{b_{2}b_{3}}{a_{2}a_{3}} & -\frac{b_{2}b_{3}}{a_{2}a_{3}} & \frac{b_{4}}{a_{4}} & \ldots \\ 1 & \frac{b_{1}}{a_{1}} & -1 & -\frac{b_{1}}{a_{1}} & \frac{b_{3}}{a_{3}} & \frac{b_{1}b_{3}}{a_{1}a_{3}} & -\frac{b_{3}}{a_{3}} & -\frac{b_{1}b_{3}}{a_{1}a_{3}} & \frac{b_{4}}{a_{4}} & \ldots \\ 1 & -1 & -1 & 1 & \frac{b_{3}}{a_{3}} & -\frac{b_{3}}{a_{3}} & -\frac{b_{3}}{a_{3}} & \frac{b_{3}}{a_{3}} & \frac{b_{4}}{a_{4}} & \ldots \\ 1 & \frac{b_{1}}{a_{1}} & \frac{b_{2}}{a_{2}} & \frac{b_{1}b_{2}}{a_{1}a_{2}} & -1 & -\frac{b_{1}}{a_{1}} & -\frac{b_{2}}{a_{2}} & -\frac{b_{1}b_{2}}{a_{1}a_{2}} & \frac{b_{4}}{a_{4}} & \ldots \\ 1 & -1 & \frac{b_{2}}{a_{2}} & -\frac{b_{2}}{a_{2}} & -1 & 1 & -\frac{b_{2}}{a_{2}} & \frac{b_{2}}{a_{2}} & \frac{b_{4}}{a_{4}} & \ldots \\ 1 & \frac{b_{1}}{a_{1}} & -1 & -\frac{b_{1}}{a_{1}} & -1 & -\frac{b_{1}}{a_{1}} & 1 & \frac{b_{1}}{a_{1}} & \frac{b_{4}}{a_{4}} & \ldots \\ 1 & -1 & -1 & 1 & -1 & 1 & 1 & -1 & \frac{b_{4}}{a_{4}} & \ldots \\ \ldots & \ldots & \ldots & \ldots & \ldots & \ldots & \ldots & \ldots & \ldots & \ldots \end{array}\right]$$

*(see* (44)*). From* (63) *we obtain an explicit formula for* $p_{ij}^{\left(n\right)}$*, namely,*

(66) $$p_{ij}^{\left(n\right)}=\frac{1}{\prod_{k=1}^{\infty }\left(1+\frac{b_{k}}{a_{k}}\right)}\sum_{l=1}^{\infty }\left[\lambda_{l}^{n}{\left(-1\right)}^{k_{l}}\left(\prod_{k\in K_{l}}\frac{b_{k}}{a_{k}}\right)\right],$$

*where* $k_{l}=|I_{i}\cap I_{l}\left|+\right|I_{j}\cap I_{l}|$ *and* $K_{l}=\left(I_{l}\cap \bar{I_{i}}\right)\cup \left(I_{j}\cap \bar{I_{l}}\right)$*. In particular, based on* (66)*, we have*

$$p_{11}^{\left(n\right)}=\frac{1}{\prod_{k=1}^{\infty }\left(1+\frac{b_{k}}{a_{k}}\right)}\sum_{i=1}^{\infty }\left[\lambda_{i}^{n}\left(\prod_{k\in I_{i}}\frac{b_{k}}{a_{k}}\right)\right],\;n\geq 1,$$

*taking the limit*

$$\lim_{n\to +\infty }p_{11}^{\left(n\right)}=\frac{1}{\prod_{k=1}^{\infty }\left(1+\frac{b_{k}}{a_{k}}\right)}=\pi_{1},$$

*where* $\pi_{1}$ *is the first component of* $\pi ={\left(\pi_{i}\right)}_{i\in N}$ *(see* (38)*). Recall that this stationary distribution is given by*

$$\pi =\frac{1}{\prod_{k=1}^{\infty }\left(1+\frac{b_{k}}{a_{k}}\right)}\cdot \left(1,\frac{b_{1}}{a_{1}},\frac{b_{2}}{a_{2}},\frac{b_{1}b_{2}}{a_{1}a_{2}},\frac{b_{3}}{a_{3}},\frac{b_{1}b_{3}}{a_{1}a_{3}},\frac{b_{2}b_{3}}{a_{2}a_{3}},\frac{b_{1}b_{2}b_{3}}{a_{1}a_{2}a_{3}},\ldots \right)$$

*and it is an eigenvector corresponding to* $\lambda_{1}=1$*.*

**Remark 3.**  *We show that with the expression on the right side of Equation* (63)*, we can associate a bounded operator in* $\ell^{2}\left(\pi \right)$*. For this purpose, define the map*

(67) $$y\to W\cdot y^{T},\;y\in \ell^{2}\left(\pi \right),$$

*where W is given by* (65) *and* $y^{T}$ *denotes the transpose of a row vector y. We prove that* (67) *defines a bounded operator. Based on* (59)*, for* $e_{i}\in \ell^{2}\left(\pi \right)$*, we have*

$$∥We_{i}^{T}{∥}_{\pi }^{2}=∥y_{\lambda_{i}}{∥}_{\pi }^{2}=\prod_{k\in I_{i}}\frac{b_{k}}{a_{k}}=\pi_{i}\cdot \prod_{k=1}^{\infty }\left(1+\frac{b_{k}}{a_{k}}\right)\leq \prod_{k=1}^{\infty }\left(1+\frac{b_{k}}{a_{k}}\right),\;i\in N.$$

*Let* $y={\left(\xi_{i}\right)}_{i\in N}\in \ell^{2}\left(\pi \right)$ *be arbitrary. Then,* $y=\sum_{i=1}^{\infty }\xi_{i}e_{i}$ *with* $\sum_{i=1}^{\infty }{\left|\xi_{i}\right|}^{2}\pi_{i}<+\infty$ *and*

(68) $$∥Wy^{T}{∥}_{\pi }^{2}=∥\sum_{i=1}^{\infty }\xi_{i}We_{i}^{T}{∥}_{\pi }^{2}\leq \sum_{i=1}^{\infty }{\left|\xi \right|}^{2}\cdot ∥We_{i}^{T}{∥}_{\pi }^{2}={∥y∥}_{\pi }^{2}\cdot \prod_{k=1}^{\infty }\left(1+\frac{b_{k}}{a_{k}}\right).$$

*The diagonal matrix* $D^{n}$ *represents a bounded operator. Its all-diagonal entries do not exceed 1 in absolute value. Thus, the product* $WD^{n}W$ *is a composition of three bounded operators in* $\ell^{2}\left(\pi \right)$*. Finally, inequality* (68) *makes it clear that the operator*

$$y\to \frac{1}{\prod_{k=1}^{\infty }\left(1+\frac{b_{k}}{a_{k}}\right)}WD^{n}W\cdot y^{T},\;y\in \ell^{2}\left(\pi \right),$$

*is a contractive one.*

*It is worth to add that the operator* x→x·W*, acting in* $l^{1}\left(N\right)$*, is unbounded. To see it, take an integer* N≥2 *and* $i≔1+\sum_{k=1}^{N}2^{k-1}$*. Then,* $I_{i}=\left\{1,2,\ldots ,N\right\}$*; therefore,* $|I_{i}|=N$*. Moreover,* $∥e_{i}{∥}_{l^{1}\left(N\right)}=1$*, for any N, and from* (45) *we obtain*

$${∥e_{i}\cdot W∥}_{l^{1}\left(N\right)}={∥x_{\lambda_{i}}∥}_{l^{1}\left(N\right)}=2^{N}\cdot \prod_{k=N+1}^{\infty }\left(1+\frac{b_{k}}{a_{k}}\right)\geq 2^{N}\to +\infty ,$$

*as* N→+∞*. Hence, W cannot be bounded, as an operator, in* $l^{1}\left(N\right)$*.*

## 4. Some Additional Results

### 4.1. Computation of $W^{-1}$

The matrix *W*, determined by (65), as well as *W* is Section 2, is a special case of the matrix $\tilde{W}$ defined by (70). So if, in $\tilde{W}$, we substitute $c_{k}=b_{k}/a_{k}$, we will obtain *W*. Therefore, we consider this more general case here.

We assume that ${\left(c_{k}\right)}_{k\in N}$ is a sequence of positive real numbers satisfying the summability condition $\sum_{k=1}^{+\infty }c_{k}<+\infty$. Then, the product $\prod_{k=1}^{+\infty }\left(1+c_{k}\right)$ converges and

(69) $$c≔\prod_{k=1}^{+\infty }\left(1+c_{k}\right)=\sum_{i=1}^{+\infty }\left(\prod_{k\in I_{i}}c_{k}\right)<+\infty$$

(see [17] (Problems 3.8.3, 3.8.26)). Equivalently, the right hand side of (69) may be written as $\sum_{I_{i}\subset N}\left(\prod_{k\in I_{i}}c_{k}\right)$, where the sum is over all finite subsets of N.

Based on the sequence ${\left(c_{k}\right)}_{k\in N}$, we define an infinite matrix $\tilde{W}$ as

(70) $$\tilde{W}=\left[{\tilde{w}}_{i,j}\right],\;{\tilde{w}}_{i,j}={\left(-1\right)}^{\left|I_{i}\cap I_{j}\right|}\cdot \prod_{k\in \bar{I_{i}}\cap I_{j}}c_{k},\;i,j\in N,$$

where $I_{i}$, $I_{j}$ are representation sets of *i* and *j*, respectively. In a more explicit form, its first eight rows are

$$\tilde{W}=\left[\begin{array}{cccccccccc} 1 & c_{1} & c_{2} & c_{1}c_{2} & c_{3} & c_{1}c_{3} & c_{2}c_{3} & c_{1}c_{2}c_{3} & c_{4} & \ldots \\ 1 & -1 & c_{2} & -c_{2} & c_{3} & -c_{3} & c_{2}c_{3} & -c_{2}c_{3} & c_{4} & \ldots \\ 1 & c_{1} & -1 & -c_{1} & c_{3} & c_{1}c_{3} & -c_{3} & -c_{1}c_{3} & c_{4} & \ldots \\ 1 & -1 & -1 & 1 & c_{3} & -c_{3} & -c_{3} & c_{3} & c_{4} & \ldots \\ 1 & c_{1} & c_{2} & c_{1}c_{2} & -1 & -c_{1} & -c_{2} & -c_{1}c_{2} & c_{4} & \ldots \\ 1 & -1 & c_{2} & -c_{2} & -1 & 1 & -c_{2} & c_{2} & c_{4} & \ldots \\ 1 & c_{1} & -1 & -c_{1} & -1 & -c_{1} & 1 & c_{1} & c_{4} & \ldots \\ 1 & -1 & -1 & 1 & -1 & 1 & 1 & -1 & c_{4} & \ldots \\ \ldots & \ldots & \ldots & \ldots & \ldots & \ldots & \ldots & \ldots & \ldots & \ldots \end{array}\right].$$

In case the sequence ${\left(c_{k}\right)}_{k\in N}$ is finite, e.g., it consists of $c_{1}$ and $c_{2}$, then

$$\tilde{W}=\left[\begin{array}{cccc} 1 & c_{1} & c_{2} & c_{1}c_{2}\\ 1 & -1 & c_{2} & -c_{2}\\ 1 & c_{1} & -1 & -c_{1}\\ 1 & -1 & -1 & 1 \end{array}\right]$$

For a given set *X*, let P(X) denote its power set, i.e., the set consisting of all subsets of *X*. The symmetric difference of *A* and *B* is defined as

$$A△B≔\left(A\cap \bar{B}\right)\cup \left(\bar{A}\cap B\right),\;A,B\in P\left(X\right).$$

It is well known that (P(X),△) is an (abelian) group (see [20] (p. 10)). The empty set is the neutral element of this group and every element of P(X) equals its own inverse. More precisely, it means that (A△B)△C=A△(B△C), A△∅=A and A△A=∅ for any A,B,C∈P(X). Because (P(X),Δ) is a group, for any fixed A∈P(X), the map f:P(X)→P(X), defined by

(71) f(B)=B△A

is a bijection (see [20] (p. 53)). This means P(X)={f(B):B∈P(X)}.

If *X* is finite and non-empty, say |X|=n, where n≥1, then

(72) $$\sum_{I\in P(X)}{\left(-1\right)}^{\left|I\right|}=0$$

(see, e.g., [16] (p. 201)). It follows from ∑k=0nnk(−1)k=0, which is a consequence of the binomial expansion (a+b)n=∑k=0nnkan−kbk, if we put a=1 and b=−1. For example, take X={1,2}. Then, P(X)={∅,{1},{2},{1,2}} and

$${\left(-1\right)}^{\left|\emptyset \right|}+{\left(-1\right)}^{\left|\{1\}\right|}+{\left(-1\right)}^{\left|\{2\}\right|}+{\left(-1\right)}^{\left|\{1,2\}\right|}=1-1-1+1=0.$$

We will use (71) and (72) in the proof of the following lemma, where $I=[\delta_{i,j}]$ denotes the identity matrix; i.e., $\delta_{i,i}=1$ and $\delta_{i,j}=0$ for i≠j.

**Lemma 5.**  *Let* $\tilde{W}$ *be the matrix defined by* (70)*. Then* ${\tilde{W}}^{2}=c\cdot I$*, which implies*

(73) $${\tilde{W}}^{-1}=c^{-1}\cdot \tilde{W},$$

*where c is given by* (69)*.*

**Proof.**  Denote $V=\left[v_{i,j}\right]={\tilde{W}}^{2}$. First, we compute $v_{1,1}$. Recall that $I_{1}=\emptyset$ and $\bar{I_{1}}=N$; hence, ${\tilde{w}}_{1,j}=\prod_{k\in I_{j}}c_{k}$ and ${\tilde{w}}_{j,1}=1$, for any j∈N. Then, from (69) we get

$$v_{1,1}=\sum_{j=1}^{\infty }{\tilde{w}}_{1,j}{\tilde{w}}_{j,1}=\sum_{j=1}^{\infty }\left(\prod_{k\in I_{j}}c_{k}\right)=c.$$

For i≥2 we have

(74) $$v_{i,i}=\sum_{j=1}^{\infty }{\tilde{w}}_{i,j}{\tilde{w}}_{j,i}=\sum_{j=1}^{\infty }\left[\left({\left(-1\right)}^{|I_{i}\cap I_{j}|}\prod_{k\in \bar{I_{i}}\cap I_{j}}c_{k}\right)\left({\left(-1\right)}^{|I_{j}\cap I_{i}|}\prod_{k\in \bar{I_{j}}\cap I_{i}}c_{k}\right)\right].$$

Notice that in (74) the powers of −1 cancel each other out because

$${\left(-1\right)}^{|I_{i}\cap I_{j}|}\cdot {\left(-1\right)}^{|I_{j}\cap I_{i}|}={\left(-1\right)}^{2|I_{i}\cap I_{j}|}=1.$$

Moreover, $\left(\bar{I_{i}}\cap I_{j}\right)\cup \left(\bar{I_{j}}\cap I_{i}\right)=I_{i}△I_{j}$. In other words, it is the symmetric difference of $I_{i}$ and $I_{j}$. The set $I_{i}$ is fixed, so by (71) the map $I_{j}\to I_{i}△I_{j}$ is a bijection. As a result, (74) takes the form

(75) $$v_{i,i}=\sum_{j=1}^{\infty }\left(\prod_{k\in I_{i}△I_{j}}c_{k}\right)=\sum_{j^{\prime }=1}^{\infty }\left(\prod_{k\in I_{j^{\prime }}}c_{k}\right)=c.$$

Now we will prove that $v_{i_{1},i_{2}}=0$, if $i_{1}\neq i_{2}$. So let $i_{1}$, $i_{2}$ be fixed and assume $i_{1}\neq i_{2}$. Then $I_{i_{1}}\neq I_{i_{1}}$, which implies $I_{i_{1}}△I_{i_{2}}\neq \emptyset$, or equivalently

(76) $$|I_{i_{1}}△I_{i_{2}}|\geq 1.$$

Based on (70), we have

(77) $$v_{i_{1},i_{2}}=\sum_{j=1}^{\infty }{\tilde{w}}_{i_{1},j}{\tilde{w}}_{j,i_{2}}=\sum_{j=1}^{\infty }\left[\left({\left(-1\right)}^{|I_{j}\cap I_{i_{1}}|}\prod_{k\in I_{j}\cap \bar{I_{i_{1}}}}c_{k}\right)\left({\left(-1\right)}^{|I_{j}\cap I_{i_{2}}|}\prod_{k\in \bar{I_{j}}\cap I_{i_{2}}}c_{k}\right)\right].$$

Let $P_{1}\left(N\right)$ denote the family of all finite subsets of N. The set $P_{1}\left(N\right)$ is countable, as a countable sum of finite sets. Then, define the map $g:P_{1}\left(N\right)\to P_{1}\left(N\right)$ as follows:

(78) $$g\left(I_{j}\right)≔\left(I_{j}\cap \bar{I_{i_{1}}}\right)\cup \left(\bar{I_{j}}\cap I_{i_{2}}\right),\;\left|I_{j}\right|<\infty .$$

Equivalently, the set $g(I_{j})$ can be written in the form

$$g\left(I_{j}\right)=\left(I_{j}\cup I_{i_{2}}\right)\setminus \left(I_{j}\cap I_{i_{1}}\right)=\left(I_{j}\cap \bar{I_{i_{1}}}\cap \bar{I_{i_{2}}}\right)\cup \left(I_{i_{2}}\setminus \left(I_{i_{1}}\cap I_{i_{2}}\cap I_{j}\right)\right).$$

The assumption $i_{1}\neq i_{2}$ means that *g* is not a bijection. To see it, define the relation

$$I_{j_{1}}∼I_{j_{2}}\;\Leftrightarrow \;g\left(I_{j_{1}}\right)=g\left(I_{j_{2}}\right),$$

where *g* is given by (78). It is straightforward to verify that it is an equivalence relation on $P_{1}\left(N\right)$. Moreover, the equivalence class of $I_{j}$ equals

(79) $$\left[I_{j}\right]=\left\{S_{I}=:\left(I_{j}\cap \bar{\left(I_{i_{1}}△I_{i_{2}}\right)}\right)\cup I:I\subset I_{i_{1}}△I_{i_{2}}\right\}.$$

Therefore, from (76), we obtain

$$\left|[I_{j}]\right|=2^{\left|I_{i_{1}}△I_{i_{2}}\right|}\geq 2,$$

which proves that *g* is not injective. Furthermore, the set $S_{I}$, in (79), can be written as a sum of three mutually disjoint sets, namely,

$$S_{I}=\left(I_{j}\cap \bar{I_{i_{1}}}\cap \bar{I_{i_{1}}}\right)\cup \left(I_{j}\cap I_{i_{1}}\cap I_{i_{2}}\right)\cup I.$$

We will show that $g\left(S_{I}\right)=g\left(I_{j}\right)$ for any $I\subset I_{i_{1}}△I_{i_{2}}$. Based on (78), the set $g(S_{I})$ can be written as $S_{I}^{\prime }\cup S_{I}^{\prime \prime }$, where

$$S_{I}^{\prime }=\left[\left(I_{j}\cap \bar{I_{i_{1}}}\cap \bar{I_{i_{1}}}\right)\cup \left(I_{j}\cap I_{i_{1}}\cap I_{i_{2}}\right)\cup I\right]\cap \bar{I_{i_{1}}}=\left(I_{j}\cap \bar{I_{i_{1}}}\cap \bar{I_{i_{1}}}\right)\cup \left(I\cap \bar{I_{i_{1}}}\right)$$

and

$$S_{I}^{\prime \prime }=\left[\left(\bar{I_{j}}\cup I_{i_{1}}\cup I_{i_{2}}\right)\cap \left(\bar{I_{i_{1}}}\cup \bar{I_{i_{2}}}\cup \bar{I_{j}}\right)\cap \bar{I}\right]\cap I_{i_{2}}=\bar{I}\cap I_{i_{2}}\cap \left(\bar{I_{i_{1}}}\cup \bar{I_{j}}\right).$$

Because $I\subset I_{i_{1}}△I_{i_{2}}$, we have $I\cap \bar{I_{i_{1}}}=I\cap I_{i_{2}}$ and

$$I_{i_{2}}\cap \left(\bar{I_{i_{1}}}\cup \bar{I_{j}}\right)=I_{i_{2}}\cap \bar{\left(I_{i_{1}}\cap I_{j}\right)}=I_{i_{2}}\setminus \left(I_{i_{1}}\cap I_{i_{2}}\cap I_{j}\right).$$

Hence, we get

$$\left(I\cap I_{i_{2}}\right)\cup \left[\bar{I}\cap \left(I_{i_{2}}\setminus \left(I_{i_{1}}\cap I_{i_{2}}\cap I_{j}\right)\right)\right]=I_{i_{2}}\setminus \left(I_{i_{1}}\cap I_{i_{2}}\cap I_{j}\right),$$

and as a result,

(80) $$g\left(S_{I}\right)=S_{I}^{\prime }\cup S_{I}^{\prime \prime }=\left(I_{j}\cap \bar{I_{i_{1}}}\cap \bar{I_{i_{2}}}\right)\cup \left(I_{i_{2}}\setminus \left(I_{i_{1}}\cap I_{i_{2}}\cap I_{j}\right)\right)=g\left(I_{j}\right).$$

Thanks to (80), we can write (77) in the form

(81) $$v_{i_{1},i_{2}}=\sum_{g(I_{j})}\left[\left(\prod_{k\in g(I_{j})}c_{k}\right)\cdot \Gamma_{I_{j}}\right],$$

where *g* is given by (78) and

$$\Gamma_{I_{j}}=\sum_{I\subset I_{i_{1}}△I_{i_{2}}}{\left(-1\right)}^{|[\left(I_{j}\cap \bar{I_{i_{1}}△I_{i_{2}})}\cup I\right]\cap I_{i_{1}}|+|[\left(I_{j}\cap \bar{I_{i_{1}}△I_{i_{2}})}\cup I\right]\cap I_{i_{2}}|}.$$

However, notice that

$$\left[\left(I_{j}\cap \bar{I_{i_{1}}△I_{i_{2}}}\right)\cup I\right]\cap I_{i_{1}}=\left(I\cap I_{i_{1}}\right)\cup \left(I_{i_{1}}\cap I_{i_{2}}\cap I_{j}\right),$$

and similarly

$$\left[\left(I_{j}\cap \bar{I_{i_{1}}△I_{i_{2}}}\right)\cup I\right]\cap I_{i_{2}}=\left(I\cap I_{i_{2}}\right)\cup \left(I_{i_{1}}\cap I_{i_{2}}\cap I_{j}\right).$$

Then, from (72), we have

$$\Gamma_{I_{j}}=\sum_{I\subset I_{i_{1}}△I_{i_{2}}}{\left(-1\right)}^{|I\cap I_{i_{1}}|+|I\cap I_{i_{2}}|}=\sum_{I\subset I_{i_{1}}△I_{i_{2}}}{\left(-1\right)}^{\left|I\right|}=0.$$

Taking into account (81), we finally obtain

(82) $$v_{i_{1},i_{2}}=0,\;if\;i_{1}\neq i_{2}.$$

This completes the proof. □

### 4.2. Computation of $\prod_{k\in J}\left(c_{k}+d_{k}\right)$

Suppose *J* is a finite subset of N. It is straightforward to check that the product $\prod_{k\in J}\left(c_{k}+d_{k}\right)$, where $c_{k},d_{k}\in R$, can be written in the following way:

(83) $$\prod_{k\in J}\left(c_{k}+d_{k}\right)=\sum_{I\subset J}\left(\prod_{k\in I}c_{k}\cdot \prod_{k\in \bar{I}\cap J}d_{k}\right).$$

The above sum runs over all subsets *I* of *J*, so there are $2^{\left|J\right|}$ terms in the expression on the right hand side of (83) (see [20] (p. 12)). For example, for J={1,2}, we have

$$\prod_{k\in \{1,2\}}\left(c_{k}+d_{k}\right)=c_{1}c_{2}+c_{1}d_{2}+c_{2}d_{1}+d_{1}d_{2},$$

and the corresponding subsets of *J* are {1,2}, {1}, {2} and *∅*, respectively.

Similarly to (83), by putting $-d_{k}$ in place of $d_{k}$, we would obtain

(84) $$\prod_{k\in J}\left(c_{k}-d_{k}\right)=\sum_{I\subset J}\left({\left(-1\right)}^{|\bar{I}\cap J|}\cdot \prod_{k\in I}c_{k}\cdot \prod_{k\in \bar{I}\cap J}d_{k}\right),$$

where, again, the sum is over all subsets of *J*. For example, consider $\prod_{k\in \{1,2\}}\left(c_{k}-d_{k}\right)$. Then,

$$\prod_{k\in \{1,2\}}\left(c_{k}-d_{k}\right)=c_{1}c_{2}-c_{1}d_{2}-c_{2}d_{1}+d_{1}d_{2},$$

which can be rewritten as

$${\left(-1\right)}^{\left|\{1,2\}\right|}c_{1}c_{2}+{\left(-1\right)}^{\left|\{1\}\right|}c_{1}d_{2}+{\left(-1\right)}^{\left|\{2\}\right|}c_{2}d_{1}+{\left(-1\right)}^{\left|\emptyset \right|}d_{1}d_{2}.$$

It turns out that if *J* is infinite and the product $\prod_{k\in J}\left(c_{k}+d_{k}\right)$ converges, then (83) also holds true, so for J=N, it would take the form

$$\prod_{k=1}^{\infty }\left(c_{k}+d_{k}\right)=\sum_{I\subset N}\left(\prod_{k\in I}c_{k}\cdot \prod_{k\in \bar{I}\cap N}d_{k}\right).$$

We will prove it in the following lemma.

**Lemma 6.**  *Assume J is an infinite subset of* N *and let* ${\left(c_{k}\right)}_{k\in J}$*,* ${\left(d_{k}\right)}_{k\in J}$ *be sequences of positive numbers such that the products* $\prod_{k\in J}\left(c_{k}+d_{k}\right)$*,* $\prod_{k\in J}d_{k}$ *converge. Then,*

(85) $$\prod_{k\in J}\left(c_{k}+d_{k}\right)=\sum_{I\subset J}\left(\prod_{k\in I}c_{k}\cdot \prod_{k\in \bar{I}\cap J}d_{k}\right),$$

*where the sum is taken over all finite subsets of J.*

**Proof.**  Because the product $\prod_{k\in J}\left(c_{k}+d_{k}\right)$ converges, then

$$\prod_{k\in J}\left(c_{k}+d_{k}\right)=\lim_{n\to +\infty }\prod_{k\in J\cap S_{n}}\left(c_{k}+d_{k}\right),$$

where $S_{n}=\left\{1,2,\ldots ,2^{n}\right\}$. The set $J\cap S_{n}$ is finite, so using (83) results in

(86) $$\prod_{k\in J}\left(c_{k}+d_{k}\right)=\lim_{n\to +\infty }\left[\sum_{I\subset J\cap S_{n}}\left(\prod_{k\in I}c_{k}\cdot \prod_{k\in \bar{I}\cap J\cap S_{n}}d_{k}\right)\right],$$

where the sum is over all subsets of $J\cap S_{n}$ (see also [17] (Problem 3.8.26)). But the product $\prod_{k\in J}d_{k}$ converges, so

$$\lim_{n\to +\infty }\prod_{k\in \bar{I}\cap J\cap S_{n}}d_{k}=\prod_{k\in \bar{I}\cap J}d_{k}.$$

All terms on the right side of (86) are non-negative, so taking the limit, we obtain (85). □

## Data Availability

No new data were created or analyzed in this study.
